# Microfluidic‐Driven Lipid Nanoparticles for Improved miRNA Delivery via Endo‐Lysosomal Trafficking Optimization

**DOI:** 10.1002/advs.202519225

**Published:** 2026-02-06

**Authors:** Alicja Kosik‐Kozioł, Michał Pruchniewski, Daniel Rybak, Piotr Jenczyk, Karolina Zakrzewska, Magdalena Bartolewska, Sławomir Błoński, Paweł Nakielski, Filippo Pierini

**Affiliations:** ^1^ Department of Biosystems and Soft Matter Institute of Fundamental Technological Research Polish Academy of Sciences Warsaw Poland; ^2^ Department of Nanobiotechnology Institute of Biology Warsaw University of Life Sciences Warsaw Poland; ^3^ Department of Mechanics of Materials Institute of Fundamental Technological Research Polish Academy of Sciences Warsaw Poland

**Keywords:** endo‐lysosomal trafficking, lipid nanoparticles, LNP post‐processing, microfluidics, miRNAs‐Cy3

## Abstract

This study investigates the influence of post‐processing techniques on lipid nanoparticles (LNPs) designed for miRNA delivery in in vitro transfection models. We compared blank and miRNA‐loaded LNPs (LNP‐miRNA) in terms of size, polydispersity index, zeta potential, electrophoretic mobility, and conductivity. miRNA encapsulation increases lipid particle size by 43.6%, due to structural rearrangements. Post‐processing methods, including sonication, filtration, dialysis, and thermal treatment, significantly alter particle characteristics. Sonication and filtration decrease particle size and improve uniformity, enhancing colloidal stability. Dialysis further refines the particle size but decreases its electrophoretic mobility. Non‐dialyzed, sonicated, and filtered LNP‐miRNA samples demonstrate the most favorable electrokinetic profile, maintaining low conductivity (0.003 mS/cm) and high electrophoretic mobility (3.16 ± 0.22 µm cm/V·s), suggesting optimal stability for gene delivery. Zeta potential measurements show that sonication and filtration increase the surface charge of LNP‐miRNA formulations from +18.9 to +29.3 mV, enhancing colloidal stability, while dialysis reduces it to +1.9 mV. Although sonicated and filtered LNP‐miRNA samples exhibited more favorable physicochemical properties, the dialyzed formulations modulate intracellular trafficking, resulting in earlier intracellular availability and prolonged persistence of delivered miRNA. This work establishes a framework for optimizing non‐viral miRNA delivery by showing how post‐processing shapes LNP stability and transfection performance.

## Graphical abstract

This study investigates the impact of various post‐processing methods (filtration, sonication, dialysis, and heating) on lipid nanoparticles produced using microfluidics. The nanoparticles are designed for efficient delivery of miRNA into cells. The work highlights how processing conditions influence nanoparticle stability and quality, providing insights into optimizing formulations for effective intracellular transfection. In particular, the study investigates how post‐processing–dependent changes in LNP properties influence endo‐lysosomal trafficking and the intracellular redistribution of delivered miRNA.

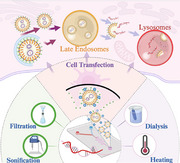

## 1 Introduction

MicroRNAs (miRNAs) are small, non‐coding RNA molecules that play an important role in regulating gene expression after transcription, and are especially involved in controlling biological processes related to inflammation [1]. They play key roles in all major biological and physiological processes, including development, morphogenesis, and skin homeostasis [2, 3]. In the skin, miRNAs influence cell proliferation, differentiation, immune regulation, and wound healing [2]. As a result, miRNA dysregulation is extensively investigated in various skin disorders, such as atopic dermatitis, psoriasis, and skin tumors [4, 5, 6]. The success of mRNA‐based vaccines against SARS‐CoV‐2 has renewed interest in RNA‐based therapeutics, including those targeting or utilizing miRNAs [7].

Despite being promising, miRNA‐based therapies face several significant challenges, such as limited tissue‐specific delivery, unintended gene regulation, and the potential for immune activation [8, 9]. The selection of an appropriate delivery vehicle is crucial for ensuring the successful administration of miRNAs [10]. An ideal vector should overcome both extracellular and intracellular barriers, shield miRNAs from enzymatic degradation, promote efficient cellular uptake and endosomal escape, and exhibit low toxicity to reduce the risk of adverse effects [11].

Reported studies have shown that miRNAs can be efficiently delivered to various cell types through the use of lipid nanoparticles (LNPs), which represent a key class of delivery vehicles in the field of nucleic acid therapeutics [12]. Structurally, LNPs are composed of four primary lipid components, each contributing to their functionality: cationic lipids bind negatively charged RNA molecules [1, 13], phospholipids help stabilize the bilayer structure [14], cholesterol enhances membrane fluidity and overall particle stability [14], and polyethylene glycol (PEG)‐functionalized lipids (PEG‐lipids) extend circulation time by preventing aggregation and immune clearance [15].

Producing LNPs with consistent and predictable properties is crucial for their therapeutic use. Microfluidic systems have emerged as a cutting‐edge technology for the controlled and reproducible synthesis of these nanoparticles, offering notable advantages over traditional methods. These systems enable meticulous control over critical process parameters, including flow rates of the lipid and aqueous phases, their mixing ratios, and the reaction times, all within precisely engineered microscale channels. This fine control allows for the creation of nanoparticles with uniform size distributions and high miRNA encapsulation efficiency, ensuring that a substantial proportion of the miRNA is effectively loaded into the nanoparticles [16].

In the context of LNP production, the residual presence of ethanol and unencapsulated miRNA following formulation poses a significant challenge to nanoparticle stability [17]. Prolonged exposure to ethanol can disrupt lipid packing and compromise bilayer integrity [18], while unencapsulated miRNA may interfere with its planned applications or induce unintended biological effects [18]. Such conditions can lead to leakage of the encapsulated cargo and reduced shelf‐life, ultimately affecting therapeutic performance [17, 18].

While significant advancements have been made in optimizing LNP formulations and understanding the physics of their assembly, such as the work by Wen et al. on the influence of microfluidic mixing parameters on LNP size and encapsulation efficiency [19] and studies by Han et al. and Binici et al. on the role of ionizable lipids in LNP structure and in vivo activity [17, 18], comparatively little attention has been paid to other processes. Kimura et al. demonstrated that LNP size can be precisely tuned not only during synthesis but also through a microfluidic post‐treatment process. By incorporating baffle structures to rapidly dilute residual ethanol, they achieved enhanced size control under high flow conditions. An integrated system enabled scalable production of siRNA‐loaded LNPs, which exhibited efficient hepatocyte delivery and strong gene silencing in vivo, with no loss of activity due to post‐treatment [20].

More recent studies have underscored the importance of post‐processing in determining the stability and translational potential of LNP formulations. Vergas et al. demonstrated that dialysis is a critical purification step, showing that insufficient removal of residual ethanol can adversely affect nanoparticle stability and size uniformity, with a stronger impact than variations in flow rate ratios during the microfluidic formulation process [21]. Complementarily, Wu et al. identified tangential flow filtration as a scalable and clinically relevant standard for LNP post‐processing, enabling efficient buffer exchange while preserving key physicochemical properties, provided that filtration parameters are carefully controlled [22]. Beyond conventional purification strategies, new post‐processing approaches aim to better control the quality of LNP after formulation. Yoon et al. presented ion concentration polarization as a method that uses an electric field to remove small molecules from LNP suspensions [23]. However, direct comparisons between different post‐processing methods are still limited, and downstream processing remains a major challenge for the clinical translation of LNP‐miRNA therapeutics. The present results on low‐intensity sonication and extrusion post‐microfluidics complement Kimura et al.’s baffle dilution by adding mechanical homogenization for miRNA LNPs. It confirms Hardianto et al.’s ethanol destabilization mechanisms, as sonication reversibly disrupts aggregates, aligning with Wen et al.’s insights on mixing and Han/Binici's findings on lipid stability. Unlike solvent‐free post‐encapsulation fusion, these methods challenge the over‐reliance on filtration alone by tolerating residuals more effectively under optimized energy, thereby enhancing scalability over extrusion's shear risks.

**SCHEME 1:**
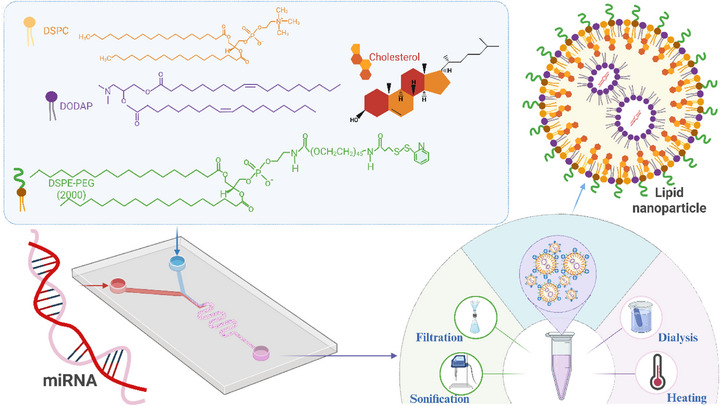
Schematic representation of LNP synthesis and post‐production processing. LNPs were formed via T‐junction microfluidic mixing of an ethanol‐dissolved lipid mixture (DODAP:DSPC:Chol:DSPE‐PEG, 10:49:40:1 mol/mol) with an acidic aqueous phase (25 mM sodium acetate, pH 4.0), with or without microRNAs. Post‐production steps included filtration, sonication, two‐step dialysis (ethanol removal and buffer exchange), and thermal incubation.

Although microfluidic LNP synthesis is commonly described as being governed by total flow rate and flow‐rate ratio, this process implicitly assumes that nanoparticle structure is fixed at the outlet of the microfluidic mixer. Recent evidence, however, suggests that LNP formation under ethanol injection conditions is a kinetically dominated process in which solvent exchange, membrane rearrangement, and fusion continue beyond the initial mixing step. In particular, the rate and pathway of ethanol removal have been shown to critically influence final LNP size and stability. Despite this, routine post‐processing steps such as dialysis, filtration, sonication, or thermal conditioning are often treated as neutral or interchangeable operations, and their mechanistic impact on LNP structure and biological performance remains insufficiently explored. To address this gap, we demonstrate that commonly used post‐processing strategies reshape the physicochemical and functional properties of microfluidic LNPs, demonstrating that post‐processing constitutes an additional kinetic window that can decouple initial particle properties from downstream transfection outcomes. In this study, we systematically investigate how post‐production techniques, including sonication, filtration, dialysis, and thermal treatment, affect the physicochemical properties and biological performance of miRNA‐loaded LNPs (LNP‐miRNA) (Scheme 1). By analyzing parameters such as particle size, polydispersity index, zeta potential, electrophoretic mobility, and conductivity in both miRNA‐loaded and blank formulations, we reveal that post‐processing significantly modulates LNPs’ stability. Finally, we examine how the resulting and processed LNP variants affect in vitro biocompatibility using L929 fibroblasts. Furthermore, we evaluate whether the developed LNP‐miRNA formulations, both dialyzed and non‐dialyzed, enable effective miRNA transfection into cells and determine the intracellular localization of the delivered miRNA. Beyond cellular internalization, intracellular trafficking and temporal persistence of delivered miRNA may critically determine the functional performance of lipid nanoparticle formulations. Our study highlights the critical yet overlooked role of post‐formulation handling in determining the therapeutic efficacy of LNP‐based RNA delivery systems, underscoring the need for improved evaluation and optimization strategies beyond initial nanoparticle synthesis.

## 2 Results and Discussion

### 2.1 Lipid Nanoparticles (LNP) Fabrication

**FIGURE 1:**
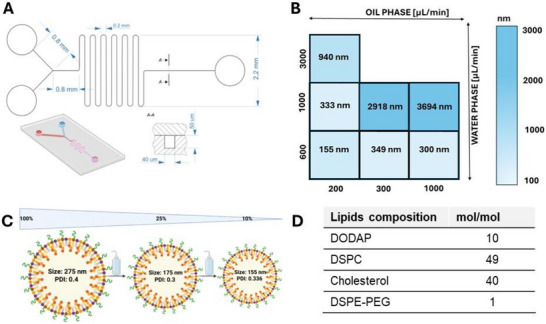
Optimization of the LNP production method. (A) Dimensions of the microfluidic channel fabricated in PDMS (width = 40 µm, height = 50 µm). (B) Optimization of the flow rate for the aqueous and oil phases, along with the resulting particle sizes measured by DLS. (C) Dependence of particle size and PDI on sample dilution in DLS measurements. (D) Lipid composition in the oil phase.

Stable encapsulation of nucleic acids, such as miRNA, in lipid nanoparticle systems requires rapid mixing of a lipid mixture containing an ionizable cationic lipid with an acidic aqueous solution containing the nucleic acids [24]. The aqueous phase is maintained at a pH below the pKa of the ionizable lipid, ensuring its protonation and positive charge, which facilitates electrostatic interactions with the negatively charged nucleic acid polymers [25]. The subsequent removal of residual ethanol results in the stable incorporation of nucleic acids into the LNP structure. To ensure high reproducibility and resolution of LNPs, microfluidic fabrication parameters were systematically optimized (Figure S1). This step involved refining the geometry of the microfluidic channels, a parameter closely linked to the flow rate of the injected solutions. We designed and fabricated a microfluidic channel with a width of 40 µm and a height of 50 µm, consisting of two inlet channels connected in a T‐junction configuration (Figure 1A).

The flow rate parameter has a critical influence on the resulting LNP diameter. We thus proceeded by tuning the solution dosing rate (aqua:oil): (i) 600:200 µL/min; (ii) 600:300 µL/min; (iii) 600:1000 µL/min; (iv) 1000:200 µL/min; (v) 1000:300 µL/min; (vi) 1000:1000 µL/min; (vii) 3000:200 µL/min (Figure 1B; Figure S2). We observed that the size of LNP particles increases with increasing flow rates of both the aqueous and oil phases. For the designed channel dimensions, 1000 µL/min represents the upper safe limit of the flow rate. Flow rates exceeding this value may cause loss of channel integrity and phase leakage at the connection points. On the other hand, the flow rate should not be too low, as it would be insufficient to ensure effective mixing of the two phases within the microfluidic channel, resulting in inefficient particle formulation. For our system, the optimal flow rate ratio was 600:200 µL/min, which enabled us to obtain LNPs with an average particle size of approximately 155 nm (PDI = 0.336). The particle size was measured by DLS with the sample diluted to 10%. For the undiluted sample (100%), the average LNP size increased to approximately 275 nm, with a PDI of 0.4 (Figure 1C). This dependence mainly arises from aggregation and interparticle interactions at higher concentrations. At higher concentrations, LNPs have a greater tendency to form aggregates, leading to the observed increase in average size. Diluting the sample reduces collision frequency and interparticle interactions, resulting in a measurement closer to the actual size of individual LNPs. After a thorough optimization of the LNP composition, we selected the lipid composition of the OIL phase, which was dissolved in ethanol as follows: DODAP/DSPC/cholesterol/DSPE‐PEG (10/49/40/1 mol/mol) (Figure 1D). The miRNA encapsulation protocol employed a microfluidic synthesis strategy, wherein an ethanolic lipid solution was rapidly mixed with an aqueous phase containing sodium acetate buffer (25 mM, pH 4.0) and negatively charged miRNA using a T‐tube mixer. The acidic pH ensured protonation of the ionizable lipid, enabling electrostatic complexation with miRNA to drive self‐assembly into LNPs. A two‐step dialysis approach was employed to ensure full biocompatibility of the final formulation. While the first step removed residual ethanol, the subsequent exchange into a physiological buffer was essential to restore pH and ionic strength conditions favorable for biological applications. To enable real‐time tracking of cellular uptake, a Cy3‐labeled Mimic Transfection Control (miRNA, 5 nmol) was dissolved directly in sodium acetate buffer to achieve a concentration of 204 nM before being mixed, allowing the fluorescent tag to be incorporated into the LNP structure during synthesis. This approach streamlined the production of functional, traceable LNPs while maintaining consistent encapsulation efficiency and minimizing solvent‐related cytotoxicity.

### 2.2 LNPs Morphology and Size

**FIGURE 2:**
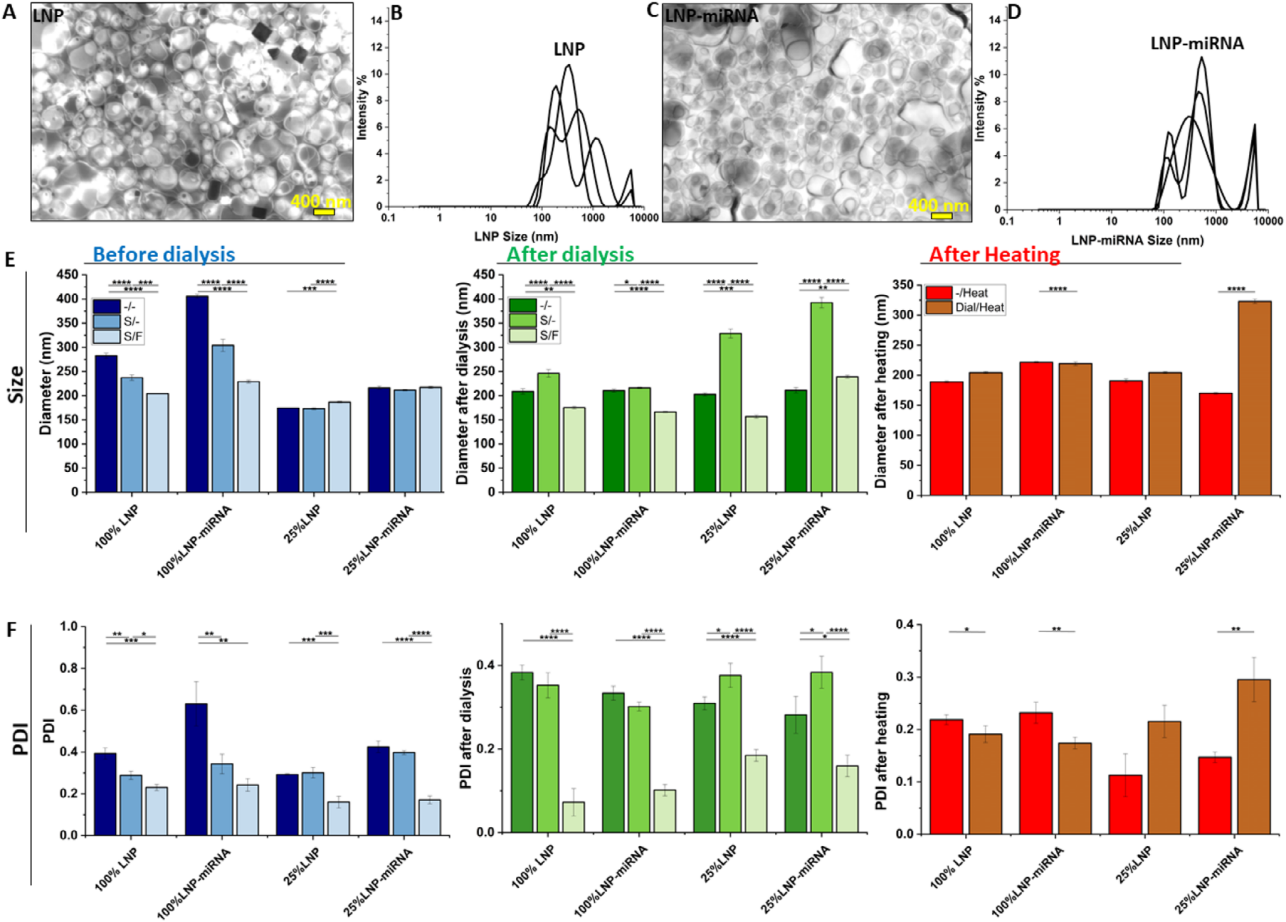
Characterization of the physicochemical properties of LNPs and LNP‐miRNA. (A) TEM image of LNPs; (B) size distribution of LNPs; (C) TEM image of LNP‐miRNA; (D) size distribution of LNP‐miRNA; (E) particle size comparison of LNPs and LNP‐miRNA depending on sample concentration (100%, 25%) and post‐processing steps: “native” before dialysis (blue), after dialysis (green), and after heating (red), neither sonificated nor filtrated (‐/‐), sonificated (S/‐), sonificated and filtrated (S/F).

STEM imaging confirmed that both LNPs and LNP‐miRNA exhibited spherical morphology with smooth surfaces (Figure 2A,C). Quantitative analysis via DLS revealed distinct size profiles: LNPs displayed a mean diameter of 282.6 ± 5.1 nm, while miRNA encapsulation increased the average size to 405.7 ± 4.0 nm (Figure 2B,D; Table S1). This size shift (∼43.6% increase) likely reflects the incorporation of the miRNA payload and associated structural rearrangements during electrostatic complexation. Both undiluted (100%) and diluted (25%) formulations of LNPs and LNP‐miRNA were analyzed via DLS to ensure measurement accuracy and mitigate concentration‐dependent artifacts. Undiluted samples (100%) reflect the “native” state of LNPs as synthesized, providing insights into their behavior in concentrated formulations, such as potential aggregation or intermolecular interactions that may arise in storage or application conditions. However, high particle concentrations can lead to multiple scattering effects or viscosity‐induced inaccuracies in DLS, which can skew hydrodynamic diameter and PDI values. Thus, diluted samples were tested to mitigate measurement artifacts. The use of ultrapure water minimized ionic interference, preserving the intrinsic physicochemical properties of LNPs while simulating dilution scenarios encountered during in vitro administration (e.g., mixing with cell culture media). Consistency between diluted and undiluted datasets confirmed structural robustness, as stable LNPs should resist aggregation or size shifts under ionic strength gradients. This dual approach balances analytical rigor (reliable DLS measurements at ideal particle density) with translational relevance, ensuring comprehensive characterization of LNP performance. DLS of 25% diluted samples revealed a mean hydrodynamic diameter of 174.3 ± 0.8 nm for LNPs, increasing to 216.1 ± 3.5 nm for LNP‐miRNA. This corresponds to a ∼24% increase in size (Δ = 41.8 nm), consistent with the structural expansion observed in undiluted formulations. The smaller absolute sizes in diluted samples reflect reduced hydration effects and interparticle interactions under low‐concentration conditions (Figure 2E, blue).

### 2.3 LNPs Post‐Fabrication Processing

Post‐fabrication processing of lipid nanoparticles is a critical step to bridge the gap between synthesis and functional application, ensuring that the physicochemical properties align with the demands of in vitro and future in vivo use. While microfluidic synthesis enables precise control over initial particle formation, subsequent steps, such as filtration, sonication, dialysis, and thermal incubation, address inherent challenges: residual solvents, particle heterogeneity, microbial contamination, and thermodynamic instability. These processes collectively refine LNPs to meet stringent criteria for size distribution, payload integrity, sterility, and physiological compatibility. Without such optimization, LNPs risk aggregation, premature miRNA release, or cytotoxicity, undermining their efficacy as delivery vehicles. Filtration ensured sterility and reduced polydispersity, though at the potential cost of losing larger LNPs (>200 nm). Sonication homogenized the particle population via ultrasonic energy, balancing aggregate disruption with thermal sensitivity through ice‐cooled, pulsed delivery. For all LNP samples, including LNP‐miRNA, both before and after dialysis, the sonication process led to a reduction in nanoparticle size. Specifically, the average diameter of 100% LNPs decreased from 282.6 ± 5.1 nm to 237.2 ± 5.4 nm, while for 100% LNP‐miRNA, the size was reduced from 405.7 ± 4.0 nm to 304.1 ± 12.8 nm. Subsequent filtration further decreased the particle sizes to 204.2 ± 0.2 nm and 229.1 ± 3.1 nm, respectively (Figure 2E, blue). Although a 0.2 µm filter is expected to retain particles larger than ∼200 nm, the hydrodynamic diameters measured by DLS for LNPs, which include their hydrated lipid shell, reflect a broad size distribution. Consequently, some particles slightly above the nominal cutoff may pass through the filter, and the average size can remain elevated due to a tail of larger particles in the distribution. Dialysis targeted biocompatibility by removing cytotoxic residuals (e.g., ethanol) and unbound miRNA, while ultracentrifugation enhanced payload specificity. After dialysis, both LNP and LNP‐miRNA samples exhibited a significant change in particle size, with reductions of approximately 26% and 48%, respectively, demonstrating the process's effectiveness in refining nanoparticle size (Figure 2E, green; Table S2). Notably, the impact of dialysis on particle size varied depending on sample concentration. For undiluted 100% LNPs, dialysis led to a size decrease of roughly one‐quarter, likely due to the removal of excess solvents or loosely bound lipids, resulting in more compact particles. Conversely, in the 25% diluted samples, dialysis caused an increase in size by approximately 16%, possibly because of changes in lipid concentration or ionic strength that promoted slight swelling or aggregation. This effect was even more pronounced in sonicated diluted samples, where particle size increased dramatically, by nearly 90% for LNPs and 86% for LNP‐miRNA. These results suggest that while sonication initially reduces particle size, the combination of dilution and dialysis can destabilize the particles, leading to significant reaggregation or structural rearrangement. However, applying filtration to these samples reduced their size to an average of 186.6 ± 1.3 nm for LNPs and 217 ± 2.1 nm for LNP‐miRNA, bringing the particle dimensions closer to those of the undiluted 100% concentration nanocarriers.

For 100% LNP, sizes are about 189.1 ± 1.7 nm after heating and 190.8 ± 3.0 nm after dialyze and heat exposure, while for 100% LNP‐miRNA, sizes are around 221.8 ± 1.1 nm with heat and decrease to 170.0 ± 1.5 nm after dialyze and heat treatment, suggesting possible structural changes or particle compaction induced by post‐treatments (Figure 2E, red; Table S3). For 25% LNP, the samples have the same size after heating, regardless of dialysis, yielding 204.5 ± 1.5 nm. However, for 25% LNP‐miRNA after dialysis combined with heat treatment, particle size increases 47.5%, which may indicate aggregation or structural changes caused by the combination of dilution, dialysis, and heat.

### 2.4 LNPs Polydispersity Index

The polydispersity index (PDI) values before dialysis for all groups, including production, sonication, and filtration steps, ranged between 0.2 and 0.4, indicating a moderate and acceptable level of nanoparticle size uniformity and distribution (Figure 2F, blue). An exception was observed for the 100% LNP‐miRNA group, which showed a higher PDI of 0.6, suggesting greater heterogeneity in particle size. However, the application of sonication in this group effectively normalized the PDI down to approximately 0.3, improving the uniformity of the nanoparticles. Further filtration reduced the PDI even more, to around 0.2, indicating a highly homogeneous nanoparticle population with a narrow size distribution (Figure 2F, blue). The combined application of dialysis, sonication, and filtration proved to be the most effective in reducing the PDI, lowering it to values around 0.1, which indicates a highly uniform nanoparticle size distribution (Figure 2F, green). Both the 100% and 25% diluted samples (LNP; LNP‐miRNA) remained within the acceptable PDI range of 0.1–0.3 after the thermal stress test. This indicates that the formulations maintained their colloidal stability under elevated temperature conditions, and no significant aggregation or instability was observed (Figure 2F, red). Collectively, these steps harmonized sterility, particle uniformity, and payload integrity, which are the key determinants of LNPs’ efficacy as miRNA delivery vectors in in vitro systems.

### 2.5 Electrophoretic Mobility of LNPs

**FIGURE 3:**
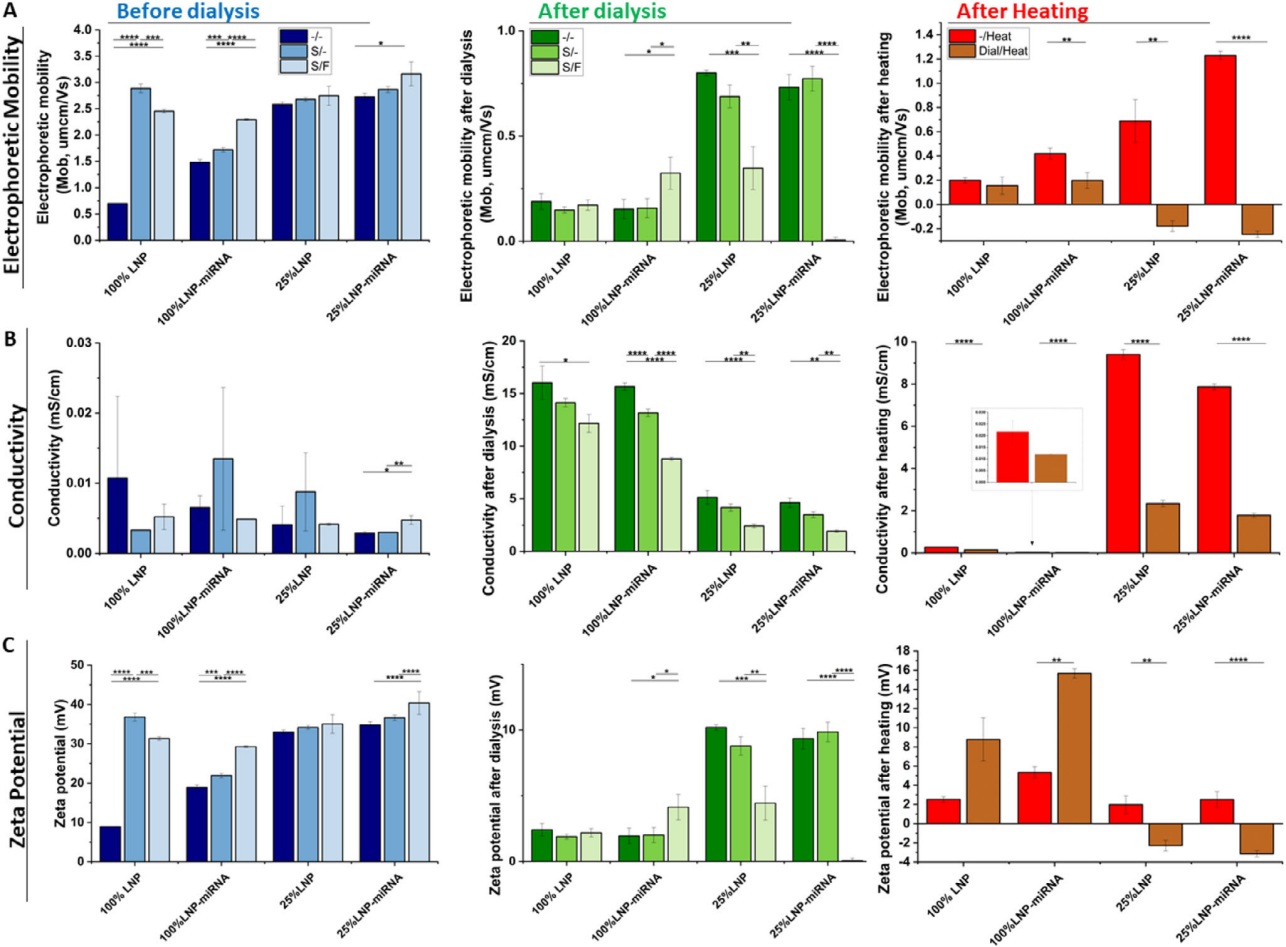
Characterization of electrokinetic properties of LNPs and LNP‐miRNA. (A) electrophoretic mobility; (B) conductivity; (C) zeta potential, depending on sample concentration (100%, 25%) and post‐processing steps: native (blue), after dialysis (green), and after heating (red), neither sonificated nor filtrated (‐/‐), sonificated (S/‐), sonificated and filtrated (S/F).

Electrophoretic mobility (EM) of DODAP‐based lipid nanoparticles reflects the velocity at which these particles move under an applied electric field, which is influenced by their surface charge and the surrounding medium [26]. Since DODAP is a cationic lipid, LNPs formulated with DODAP typically exhibit positive electrophoretic mobility values, indicating a net positive surface charge. This positive charge facilitates effective binding with negatively charged molecules such as nucleic acids (e.g., miRNA), thereby enhancing encapsulation efficiency and cellular uptake [27]. Changes in electrophoretic mobility after processes such as sonication, filtration, or dialysis can indicate alterations in surface charge density or particle stability, which are typical parameters for optimizing LNP formulations for drug delivery applications. The electrophoretic mobility of DODAP‐based LNPs typically ranges from +0.5 × 10^−8^ to +2.0 × 10^−8^ m^2^/V·s, depending on formulation specifics and the surrounding medium [26]. Before dialysis, freshly prepared 25% LNP‐miRNA samples exhibited high EM values, with the maximum value observed after sonication and filtration (3.16 ± 0.22 µm cm/V·s), indicating strong surface charge and likely high colloidal stability. This suggests efficient miRNA complexation and good dispersion stability at this stage (Figure 3A, blue). However, after dialysis, the EM of the same formulation dropped drastically to near‐neutral levels, with the lowest value recorded at 0.005 ± 0.01 µm cm/V·s after sonication and filtration. This sharp decrease may reflect the removal of unbound or weakly interacting charged species (e.g., free miRNA, excess lipids, or ions), but it may also suggest a loss in colloidal stability and surface charge density after purification (Figure 3A, green). These findings suggest that while sonication and filtration enhance charge properties and homogeneity in diluted systems, dialysis significantly reduces surface mobility, potentially impacting delivery efficiency. Therefore, formulations prior to dialysis, particularly those subjected to sonication and filtration, appear to offer the most favorable characteristics for miRNA complexation and nanoparticle stability. Following a 72 h thermal stress test at 36°C, EM values remained consistently higher for non‐dialyzed samples, both in full (100%) and diluted (25%) concentrations. For instance, 25% LNP‐miRNA heated without dialysis reached an EM of 1.22 ± 0.03 µm cm/V·s, whereas its dialyzed equivalent dropped to just −0.25 ± 0.03 µm cm/V·s. Similarly, 25% LNP without dialysis showed significantly higher mobility (0.69 ± 0.17 µm cm/V·s) than the dialyzed and heated version (−0.18 ± 0.04 µm cm/V·s) (Figure 3A, red). These negative shifts in EM values after dialysis and heating indicate a potential loss of colloidal stability and surface charge integrity. These findings strongly suggest that avoiding dialysis helps preserve the electrophoretic mobility and surface characteristics of LNPs, even under thermal stress. Elevated electrophoretic mobility in non‐dialyzed samples may be associated with enhanced colloidal stability and miRNA complexation efficiency, both of which are essential for effective delivery performance.

### 2.6 Conductivity of LNPs

Conductivity measurements for LNP suspensions typically range from 0.01 to 2.0 mS/cm, determined primarily by the ionic strength of the dispersion medium [28]. Conductivity influences the thickness of the electrical double layer and can affect zeta potential interpretation [29]. Before dialysis, the conductivity values of freshly fabricated LNPs and LNP‐miRNA complexes were relatively low across all concentrations, ranging from 0.002 to 0.01 mS/cm, depending on the formulation and treatment conditions. Notably, the highest conductivity (0.01 ± 0.01 mS/cm) was observed in 100% LNP‐miRNA after sonication, likely due to the increased dispersion of charged components in the suspension (Figure 3B, blue). Following dialysis, a substantial increase in conductivity was observed across all groups, particularly in undiluted (100%) samples. For instance, 100% LNPs and 100% LNP‐miRNA exhibited post‐dialysis conductivity values of 16.3 ± 1.6 mS/cm and 15.6 ± 0.4 mS/cm (Figure 3B, green), respectively. This significant conductivity shift caused by dialysis likely results from the replacement of organic solvents or unreacted formulation components with aqueous buffer ions during dialysis, leading to a higher concentration of freely mobile ions in solution. In diluted (25%) samples, a similar but less pronounced trend was noted: conductivity rose from 0.004–0.008 mS/cm pre‐dialysis to 2.42–5.1 mS/cm post‐dialysis, depending on the treatment. While this increase suggests effective ion exchange during dialysis, it also highlights a potential drawback: elevated ionic strength may destabilize the nanoparticle surface or alter interactions with nucleic acids, especially at lower concentrations. From a formulation stability standpoint, the most favorable conductivity values were observed in non‐dialyzed, sonicated LNPs, particularly the 25% LNP‐miRNA, which maintained moderate conductivity (0.003 mS/cm) and high electrophoretic mobility (2.86 ± 0.05 µm cm/V·s): an optimal combination for stable colloidal behavior and potential delivery performance. Dialysis introduces a substantial increase in ionic conductivity, particularly in undiluted samples, which may negatively impact surface charge characteristics and complexation efficiency with miRNA. It is important to note that the cell studies will be conducted in cell culture medium, where the miRNA addition will be further diluted, corresponding to the lower concentrations investigated here. Therefore, the superior electrokinetic properties observed in non‐dialyzed, sonicated samples at 25% concentration are especially relevant for predicting in vitro performance.

Additional conductivity measurements were performed on LNP and LNP‐miRNA samples after heating at 36°C, both with and without prior dialysis. The data further support previous findings that non‐dialyzed samples exhibit more favorable conductivity values, indicative of better nanoparticle stability. Specifically, non‐dialyzed 100% LNP and 100% LNP‐miRNA samples showed low conductivity after heating (0.28 ± 0.01 and 0.02 ± 0.04 mS/cm, respectively), consistent with earlier measurements indicating a stable ionic environment. Similarly, 25% non‐dialyzed samples maintained low conductivity (0.14 ± 0.001 for LNP and 0.01 ± 5.7 × 10^−5^ mS/cm for LNP‐miRNA), confirming that thermal treatment alone does not significantly disrupt the nanoparticle formulation (Figure 3B, red). Dialyzed and heated 25% samples exhibited markedly increased conductivity values, 9.4 ± 0.22 and 7.9 ± 0.14 mS/cm for LNP and LNP‐miRNA, respectively. After heating, these elevated conductivities suggest a higher concentration of free ions in solution, potentially impairing nanoparticle integrity and miRNA complexation. Interestingly, dialyzed and heated 25% LNP and LNP‐miRNA samples showed a similar trend to dialyzed‐only counterparts. Overall, these results confirm that avoiding dialysis better preserves nanoparticle stability under thermal stress, as reflected by lower conductivity values. Dialysis, while useful for removing impurities, appears to increase ionic conductivity and potentially compromise LNP formulation integrity, especially in concentrated samples.

### 2.7 Zeta Potential of LNPs

The zeta potential has become a standard analytical measurement for characterizing nanoparticle surfaces. It is defined as the potential at the hydrodynamic shear boundary (i.e., the slipping plane), which can provide insight into nanoparticle stability, circulation times, protein interactions, particle cell permeability, and biocompatibility [30]. For lipid nanoparticles formulated with ionizable lipids such as DODAP, the zeta potential is generally mildly positive to near neutral at physiological pH, due to the lipid's pH‐dependent ionization properties. At acidic pH values (e.g., during formulation or in endosomal environments), DODAP becomes protonated, imparting a positive zeta potential, typically in the range of +5–+60 mV [31]. This helps facilitate electrostatic complexation with negatively charged nucleic acids and promotes efficient endosomal escape [32]. Additionally, lipid PEG helps stabilize charged liposomes. Even a small amount (0.2 mol%) can prevent them from falling out of solution, allowing them to stay stable for up to 6 months instead of just 24 h [33]. Zeta potential measurements were conducted to assess the surface charge and predict the colloidal stability of both LNP and LNP‐miRNA formulations at different stages of processing (fabrication, sonication, and filtration). Immediately after fabrication, the 100% LNP and LNP‐miRNA samples exhibited relatively low zeta potential values (below 20 mV), which may indicate limited electrostatic stabilization and a higher tendency for particle aggregation. In contrast, the diluted (25%) samples showed significantly higher values, suggesting enhanced surface charge density and improved stability upon dilution. Sonication had a pronounced effect on the 100% LNP formulation, increasing the zeta potential from a low baseline to values above +35 mV, indicating a substantial improvement in nanoparticle stability. A smaller but still positive shift was observed for the 100% LNP‐miRNA group. Notably, the 25% formulations maintained or slightly increased their already high zeta potentials after sonication, confirming the stabilizing effect of this process, particularly in low‐concentration systems (Figure 3C, blue). The zeta potential values of the LNP‐miRNA formulations decreased significantly after dialysis, with values as low as +1.9 mV for undiluted samples (18.9 mV before dialysis) and 9.3 mV for 25% dilutions (34.8 mV before dialysis) (Figure 3C, green). This drop in surface charge suggests effective complexation of the negatively charged miRNA with the cationic lipids in the LNPs. Since dialysis removes unbound miRNA and lipids, the resulting low zeta potential values reflect the true surface properties of miRNA‐loaded particles and indicate successful encapsulation. Thermal stress testing at 36°C for 24 h revealed distinct differences in the surface charge behavior of various LNP formulations. Undiluted LNP and LNP‐miRNA samples maintained low but positive zeta potential values (+2.5 and +5.3 mV, respectively), suggesting moderate colloidal stability. In contrast, diluted samples without dialysis exhibited significantly higher zeta potential (+8.7 and +15.6 mV), indicating enhanced electrostatic repulsion and stability. When dialysis was applied prior to thermal exposure, the zeta potential dropped below zero (−2.26 and −3.14 mV), potentially due to the loss of cationic surface lipids or the dominant presentation of miRNA on the particle surface. These findings suggest that while thermal exposure alone does not drastically compromise surface charge, the combination of dialysis and dilution may lead to surface rearrangements that reduce stability (Figure 3C, red).

### 2.8 Binding Efficiency of LNP‐miRNA

**FIGURE 4:**
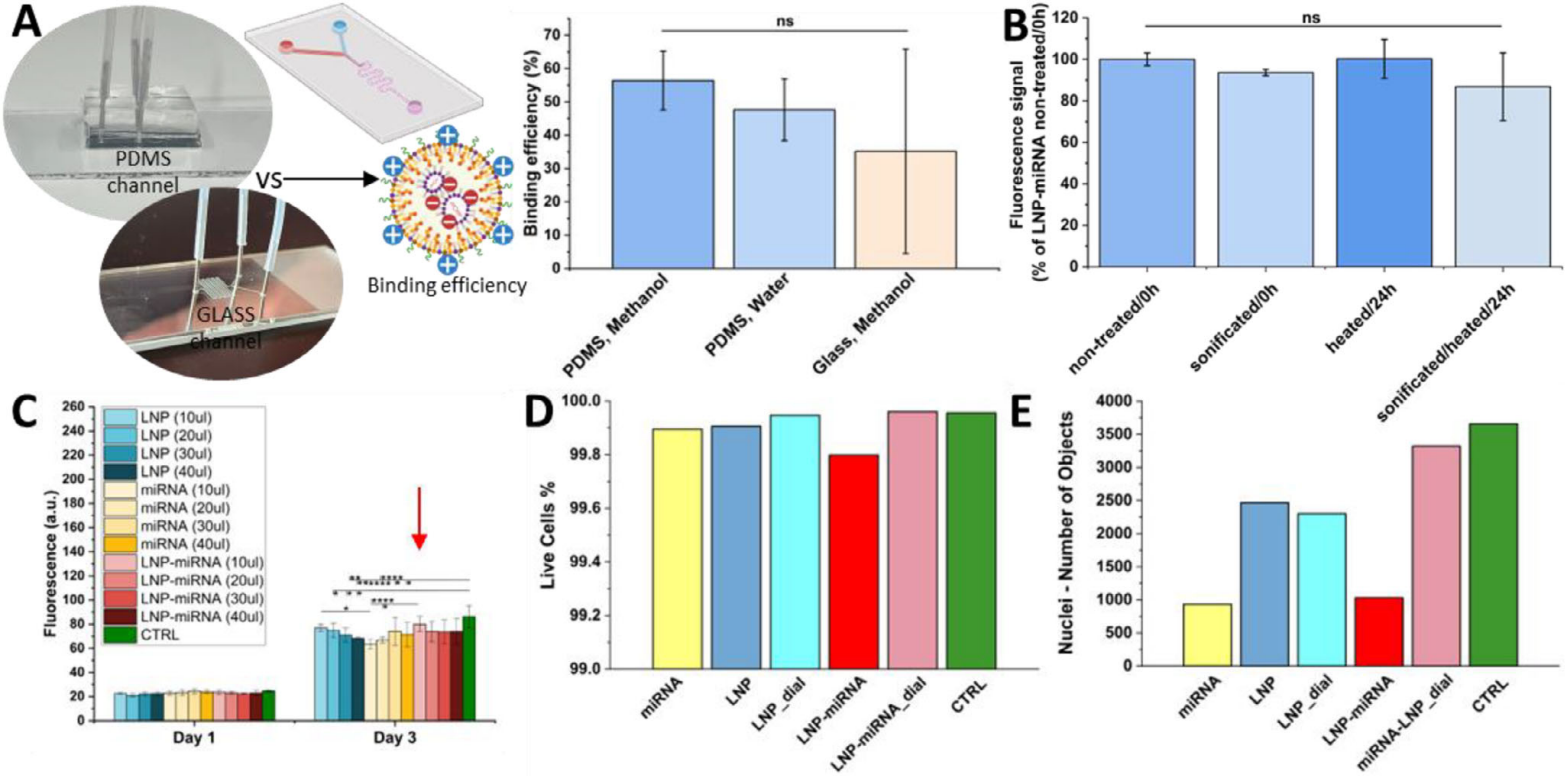
miRNA Binding efficiency and in vitro study on L929 cells. (A) Binding efficiency of miRNA‐loaded LNPs evaluated in different solvent conditions (methanol and water) and on different microfluidic channel material (PDMS and Glass); (B) Assessment of miRNA‐LNP integrity after sonification, heating, and combined sonification and heating (C) Cell proliferation analyzed by PrestoBlue assay after treatment with increasing doses of LNPs, free miRNA, and LNP‐miRNA complexes (red arrow indicates the volume selected for further studies) (n = 5 samples/group); (D) Percentage of live cells following treatment with miRNA, LNP, dialyzed LNP (LNP_dial), miRNA‐loaded LNP (LNP‐miRNA), and dialyzed miRNA‐loaded LNP (LNP‐miRNA_dial); (E) Quantification of total cell nuclei in each treatment group, including miRNA, LNP, LNP_dial, LNP‐miRNA, LNP‐miRNA_dial, and CTRL. For (E,F) Data represent mean ± SD of 9 analyzed fields from a single well (technical replicates).

In this study, we evaluated the binding efficiency of lipid nanoparticles loaded with microRNA in two different solvents: methanol and water. LNP‐miRNA complexes are widely used in nucleic acid delivery systems, and binding efficiency (defined as the percentage of miRNA successfully encapsulated or associated with LNPs) is a key parameter determining their therapeutic potential [34]. The analysis was performed in both methanol and water to assess the influence of solvent polarity and chemical environment on LNP‐miRNA interactions. Methanol, being an organic solvent, can disrupt or solubilize lipid membranes differently than water, potentially affecting the release or measurement of miRNA from the nanoparticle matrix. Based on the calculation of bound miRNA (Equation 1) and unbound miRNA (Equation 2), we calculated the miRNA binding efficiency of the developed LNP (Equation 3). Our results showed a binding efficiency of 56.4% ± 8.8% in methanol and 47.6% ± 9.3% in water (Figure 4A). These values are notably lower than those typically reported in the literature, where binding efficiencies above 80%–90% are common for optimized LNP formulations [35]. Comparisons between this work and other studies should be interpreted with caution, as many reports rely on the RiboGreen assay to estimate RNA encapsulation efficiency. In the present study, due to the use of Cy3‐labeled miRNA, encapsulation was quantified by direct fluorescence measurement of the labeled miRNA, which may yield lower apparent encapsulation values. RiboGreen‐based assays are less reliable for fluorescently labeled miRNA due to spectral dye interference and altered dye accessibility. Consistent with this, Schober et al. recently demonstrated that commonly reported encapsulation efficiency values can overestimate true RNA loading, as calculations based on RNA input frequently reveal lower efficiencies and highlight previously unreported RNA loss, particularly for small RNA molecules [36]. The relatively low efficiencies observed in our system may be attributed to partial loss of miRNA during formulation, potentially due to non‐specific adsorption or retention within the microfluidic channel system used for LNP synthesis. Although microfluidic‐based LNP fabrication offers several advantages, including high reproducibility, precise control over particle size, scalability, and compatibility with continuous‐flow systems [25], our results suggest that, in this case, it may lead to reduced miRNA encapsulation efficiency. This could result from rapid mixing dynamics or interactions between miRNA and the internal surfaces of the microfluidic device. Given that miRNA is a highly adhesive molecule with a strong tendency to adsorb to various surfaces, partial loss during loading and handling is likely to occur, particularly in microfluidic systems. To minimize such losses, all experiments were performed using low‐binding pipette tips/eppendorf tubes, as well as DNA‐free water. However, the internal surfaces of PDMS microfluidic channels cannot be readily modified without altering channel chemistry or potentially affecting LNP composition and formation. To investigate whether the surface material contributes to the observed losses, an additional microfluidic device was fabricated from glass, maintaining the same channel dimensions *(4.2.7. Preparation of Glass Microfluidic Channels)*. Additional experiments were conducted to assess miRNA binding efficiency using this glass‐based system. The binding efficiency of LNP‐miRNA measured on glass surfaces in methanol was significantly lower and exhibited markedly higher variability (35.2% ± 30.6%) compared to measurements performed in PDMS‐based systems (56.4% ± 8.8%). The large standard deviation observed for glass indicates limited measurement stability, most likely resulting from nonspecific adsorption of miRNA onto hydrophilic silica surfaces. In contrast, the use of PDMS, which is a hydrophobic material with low affinity for nucleic acids, enabled the determination of higher and substantially more reproducible binding efficiency values.

To assess the integrity of the LNP‐miRNA complex, an additional spectrophotometric analysis was performed for the LNP‐miRNA formulation before and after sonication, heating, and combined sonication and heating. Measurements were conducted immediately after treatment (0 h) and after 24 h of incubation. No substantial changes in the spectrophotometric signal were observed between the non‐treated and treated samples at either time point, indicating that neither sonication nor heating led to a pronounced loss of signal (Figure 4B). Nevertheless, the preservation of the Cy3 fluorescence signal following post‐processing steps provides indirect evidence that the labeled miRNA construct remained intact under the applied conditions. Since Cy3 is covalently conjugated to the miRNA, significant degradation or structural disruption of the miRNA would be expected to result in a decrease or alteration of the fluorescence signal.

Conventional techniques such as thin film hydration, which involves forming a dry lipid film using a rotary evaporator and subsequently hydrating it with an aqueous miRNA solution, may prove more effective for miRNA loading. While this method typically yields larger and less uniform particles, the extended hydration time and absence of micro‐scale flow channels may help preserve more of the input miRNA and improve encapsulation in certain applications.

### 2.9 Biocompatibility of LNPs

#### 2.9.1 LNPs Cytotoxicity

Subsequently, to assess the cytotoxicity of the developed LNP formulations and their varying concentrations on cell proliferation, a 3‐day quantitative PrestoBlue assay was performed. Cells in a 96‐well plate were treated with LNPs, free miRNA, or LNP‐miRNA complexes at volumes ranging from 10 to 40 µL (10 mg lipids/mL Oil phase, 204 nM miRNA) per well. Due to an approximate 50% loading efficiency of miRNA into LNPs, the free miRNA group received twice the miRNA amount theoretically compared to the LNP‐miRNA group at the same volume. The results showed a slight decreasing trend in proliferation with increasing volume in the LNP‐only group, ranging from approximately fluorescent signal 77.0 a.u. at 10 µL down to 68.3 a.u. at 40 µL; however, these differences were not statistically significant, indicating minimal cytotoxicity. In comparison, cells treated with free miRNA exhibited slightly lower overall proliferation values (ranging from 63.6 to 74.2 a.u.). However, it's important to note that this involves a double dose of miRNA compared to LNP‐miRNA, as indicated by the binding efficiency. The LNP‐miRNA complexes maintained relatively stable proliferation levels between 73.7 and 80.0 a.u. across all doses, with the minimal cytotoxicity observed at 10 µL, suggesting effective delivery with limited cytotoxic effects (Figure 4C). In contrast, the untreated control reached a proliferation level of 86.4 a.u., highlighting the potential of LNP‐miRNA to enhance cellular activity while minimizing harmful effects. Based on these findings, the 10 µL dose of LNP‐miRNA was selected for further experiments as it showed the best balance between cell viability and miRNA delivery efficiency.

#### 2.9.2 L929 Viability

Quantification of calcein‐stained cells revealed a consistently high live cell population across all groups analyzed, with negligible differences in viability observed between treatments. The percentages of live cells for miRNA‐loaded LNP (LNP‐miRNA) and dialyzed LNP‐miRNA (LNP‐miRNA_dial) were 99.79% and 99.96%, respectively, both comparable to the untreated control. These results indicate that none of the tested formulations caused significant cytotoxicity, demonstrating excellent biocompatibility under the experimental conditions. Although LNP‐miRNA_dial showed slightly higher viability than LNP‐miRNA, this difference was not statistically significant, suggesting a potentially more favorable biocompatibility profile for the dialyzed formulation (Figure 4D).

It is important to emphasize that cell viability and the number of cell nuclei (i.e., the number of cells) represent two distinct parameters. While viability reflects the proportion of living cells in the population, the number of nuclei indicates the total cell count. In this study, the presence of miRNA had the most significant impact on reducing the number of cells. This effect was observed for free miRNA, but was especially pronounced in the LNP‐miRNA group, which had noticeably fewer cells compared to its dialyzed counterpart (LNP‐miRNA_dial). This suggests that dialysis effectively removed free miRNA particles that were not encapsulated within the LNPs and that these unbound miRNA molecules may have had a negative effect on cell proliferation. Importantly, the number of cells in the LNP and LNP_dial groups did not differ substantially, indicating that dialysis does not alter the activity of the LNPs themselves (Figure 4E). In summary, unbound miRNA appeared to have the greatest influence on reducing cell numbers, and dialysis played a key role in purifying the formulations, which likely contributed to improved cellular outcomes in the dialyzed groups.

#### 2.9.3 L929 Cell Internalization

Transfection is a critical step in evaluating the functional delivery capability of lipid nanoparticle systems designed for nucleic acid transport. It involves the introduction of nucleic acids, such as microRNA, into living cells to assess whether the delivery system enables efficient intracellular uptake and subsequent release of the genetic cargo. Successful transfection is essential for eliciting the intended biological response and is therefore considered a key indicator of formulation efficacy. A fundamental aspect governing the success of this process is the electrostatic interaction between the delivery vehicle and the nucleic acid cargo. MicroRNAs carry a strong negative charge due to their phosphate backbone, whereas properly formulated LNPs are engineered to possess a net positive surface charge: typically achieved through the inclusion of cationic or ionizable lipids. This charge differential promotes electrostatic complexation between LNPs and miRNA, ensuring stable encapsulation. Additionally, the positive surface charge of LNPs enhances their interaction with the negatively charged cellular membrane, facilitating endocytosis and uptake into the cytoplasm. Once internalized, the LNPs must efficiently release their miRNA payload to allow for its biological activity within the cytoplasm or nucleus. In this study, we investigated the miRNA intracellular localization of the following formulations: miRNA, miRNA‐loaded LNPs (LNP‐miRNA), and their dialyzed counterparts (LNP‐miRNA_dial), in order to assess how physicochemical differences between the two systems affect cellular uptake and distribution. Based on our physicochemical characterization results, the LNP‐miRNA group demonstrated superior properties compared to the dialyzed LNP‐miRNA (LNP‐miRNA_dial) group.

**FIGURE 5:**
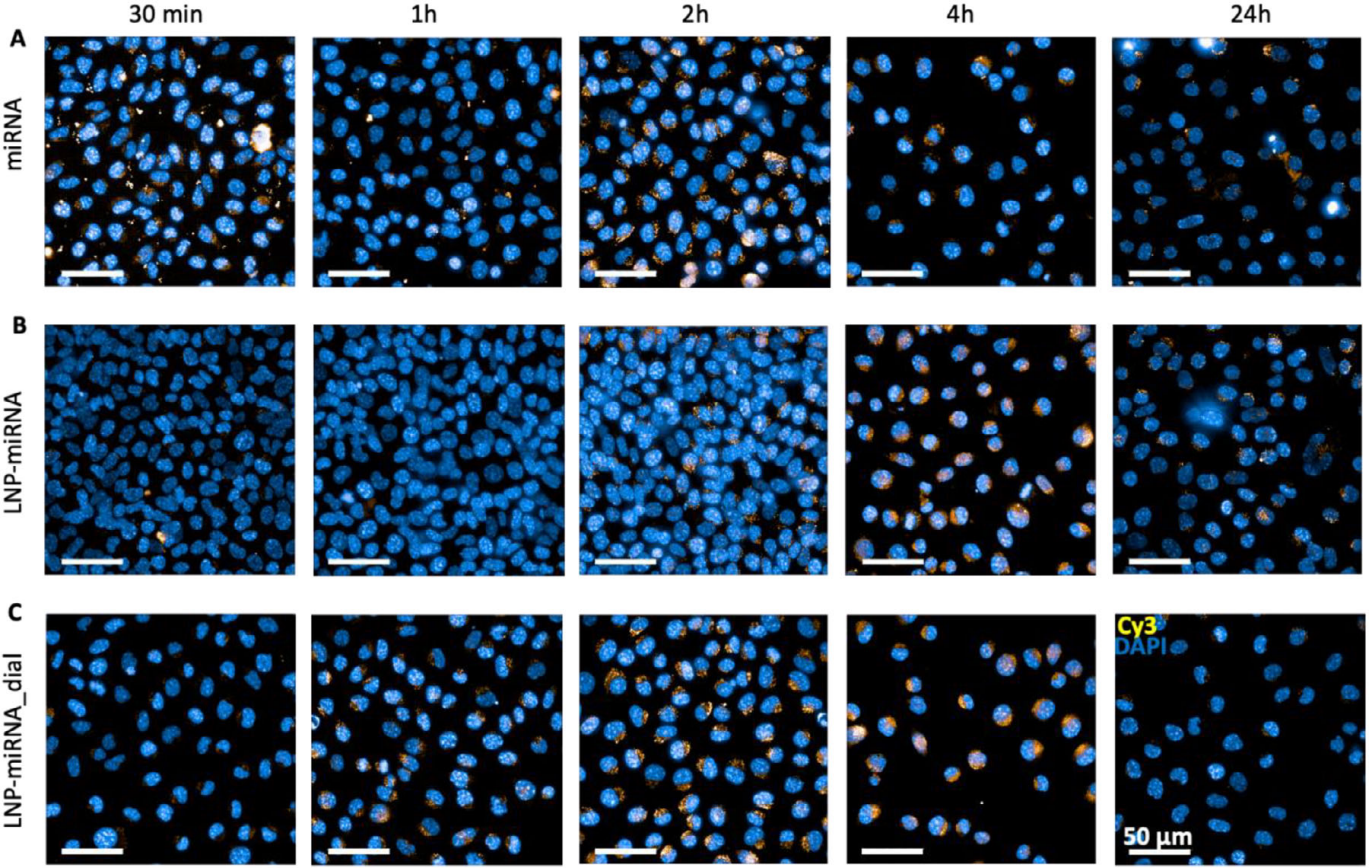
Time‐dependent intracellular localization of Cy3‐labeled miRNA relative to the cell nucleus in L929 fibroblasts. Representative confocal microscopy images showing Cy3‐labeled miRNA (yellow) and DAPI‐stained nuclei (blue) in L929 fibroblasts treated with free miRNA (A), LNP‐miRNA (B), or LNP‐miRNA_dial (C) at the indicated time points (30 min, 1 h, 2 h, 4 h, and 24 h). Cy3‐miRNA signal is predominantly cytoplasmic and localized in the perinuclear region, with formulation‐dependent differences in the timing and intensity of intracellular miRNA appearance. Scale bar: 50 µm.

For free miRNA, the Cy3 signal was readily detectable as early as 30 min post‐treatment (Figure 5A). The signal appeared predominantly as punctate and diffuse cytoplasmic fluorescence distributed around DAPI‐stained nuclei, indicating rapid cellular internalization and early cytoplasmic availability of miRNA. This spatial pattern persisted at 1 and 2 h, with Cy3 signal remaining localized primarily in the perinuclear cytoplasmic region rather than within the nucleus itself. However, the Cy3 signal was transient and markedly reduced at later time points (4 and 24 h). In contrast, for LNP‐miRNA, Cy3 signal was minimal or absent at early time points (30 min–1 h) (Figure 5B). A discernible Cy3 signal first became apparent at approximately 2 h post‐treatment and increased further at 4 h, indicating a delayed intracellular appearance compared to free miRNA. At these later time points, Cy3 fluorescence was observed mainly in the cytoplasm, with enrichment in the perinuclear region surrounding DAPI‐stained nuclei, and the signal persisted longer than in the free miRNA condition.

Notably, for LNP‐miRNA_dial, Cy3 signal was detectable earlier than for LNP‐miRNA, becoming evident at 1 h post‐treatment and increasing in intensity at 2–4 h (Figure 5C). This represents earlier intracellular detection of Cy3 compared to the non‐dialyzed LNP‐miRNA. The Cy3 signal exhibited a more homogeneous cytoplasmic distribution with pronounced localization in the perinuclear region, indicating earlier intracellular availability of miRNA relative to LNP‐miRNA. Across all treatment conditions, Cy3 signal remained predominantly cytoplasmic and nuclear‐proximal, with no clear accumulation within the DAPI‐stained nuclear compartment. At 24 h post‐treatment, Cy3 signal intensity decreased across all groups, consistent with miRNA degradation, dilution during cell division, or redistribution within the cytoplasm.

#### 2.9.4 Endo‐Lysosomal Trafficking and Endosomal Escape of miRNA

**FIGURE 6:**
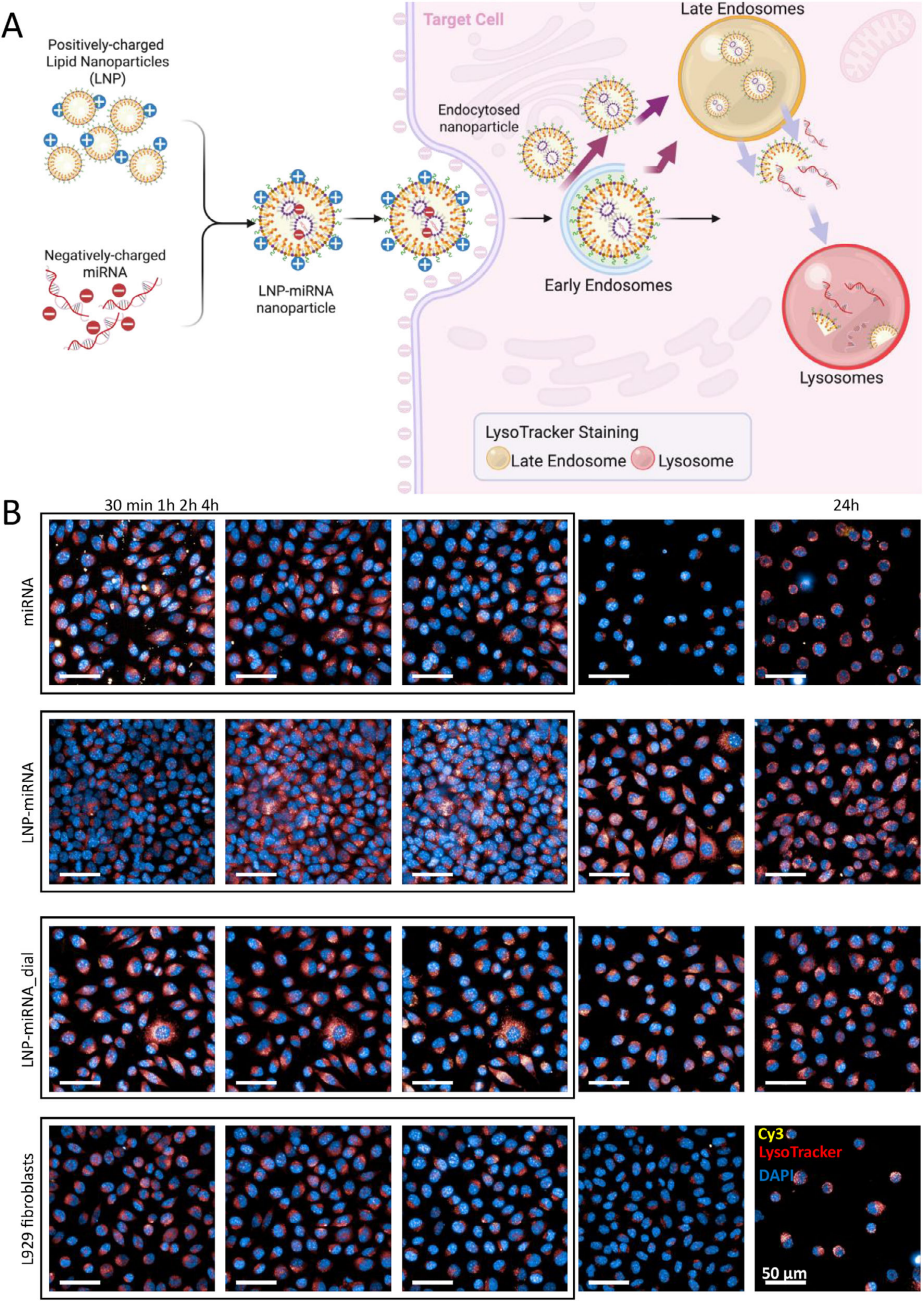
Endo‐lysosomal trafficking and intracellular redistribution of miRNA in L929 fibroblasts. (A) Schematic illustration of miRNA complexation with lipid nanoparticles (LNPs), cellular uptake via endocytosis, and subsequent trafficking through early and late endosomal and lysosomal compartments visualized using LysoTracker staining. (B) Representative confocal microscopy images of L929 fibroblasts treated with free miRNA, LNP‐miRNA, or dialyzed LNP‐miRNA_dial and imaged at 30 min, 1 h, 2 h, 4 h, and 24 h post‐treatment. Cy3‐labeled miRNA is shown in yellow, LysoTracker‐stained acidic compartments in red, and nuclei (DAPI) in blue. Scale bar: 50 µm.

Efficient miRNA delivery requires not only cellular internalization of nanocarriers but also productive intracellular trafficking that enables release of the cargo into the cytoplasm (Figure 6A). In lipid‐based delivery systems, sequestration within the endo‐lysosomal pathway represents a major bottleneck that can limit functional transfection despite efficient cellular uptake. Therefore, elucidating formulation‐dependent differences in intracellular trafficking and endosomal escape is essential for understanding discrepancies in transfection performance.

The primary objective of the analysis presented in Figure 6B was to determine whether the observed differences in transfection efficiency between miRNA, LNP‐miRNA, and LNP‐miRNA_dial arise from distinct intracellular trafficking pathways rather than from differences in cellular uptake. Confocal microscopy enabled time‐resolved visualization of the spatial relationship between Cy3‐labeled miRNA, acidic endo‐lysosomal compartments, and the cell nucleus. Imaging at 30 min, 1 h, and 2 h allowed monitoring of the same groups of cells over time, which were explicitly marked by frames in the images to indicate identical regions of interest. According to the manufacturer's recommendations, cells should not be exposed to LysoTracker for longer than 2 h; therefore, endo‐lysosomal changes observed at later time points were visualized in separate fields of view rather than by prolonged LysoTracker staining of the same cells. The intracellular distribution patterns observed in Figure 6B highlight formulation‐dependent differences in miRNA processing following cellular uptake. While Cy3‐labeled miRNA remained predominantly cytoplasmic and perinuclear across all conditions, marked differences were observed in the extent and temporal evolution of LysoTracker‐positive acidic compartments. In the miRNA‐alone group (Figure S3), partial overlap between the Cy3‐miRNA signal and LysoTracker‐positive compartments was observed at early time points (30 min and 1 h), with more pronounced colocalization becoming evident from 2 h post‐treatment. By 4 h, both Cy3‐miRNA and LysoTracker signals were markedly reduced, indicating a transient intracellular presence of naked miRNA. In the free miRNA group, extensive acidic compartment accumulation became evident primarily at the 24 h time point, coinciding with a pronounced reduction of the Cy3 signal. In contrast, LNP‐miRNA treatment (Figure 6B; Figure S4) resulted in a delayed appearance of a strong Cy3‐miRNA signal, which became prominent from 2 h post‐treatment. The Cy3 signal largely overlapped with LysoTracker‐positive acidic compartments from the time of its appearance, consistent with intracellular trafficking through acidic vesicles. Notably, the Cy3‐miRNA signal persisted up to 4 h and gradually decreased by 24 h, in contrast to the faster signal loss observed for naked miRNA. For the LNP‐miRNA_dial group (Figure 6B; Figure S5), the Cy3‐miRNA signal was detectable as early as 30 min after treatment and showed substantial colocalization with LysoTracker‐positive compartments throughout the incubation period. The signal remained strong up to 4 h and declined by 24 h. The early and sustained colocalization with LysoTracker‐stained acidic vesicles suggests faster intracellular availability and altered trafficking dynamics of miRNA delivered via dialyzed LNPs compared to non‐dialyzed formulations. Together, these findings suggest that post‐synthesis dialysis of LNPs influences intracellular trafficking behavior rather than cellular entry alone.

**FIGURE 7:**
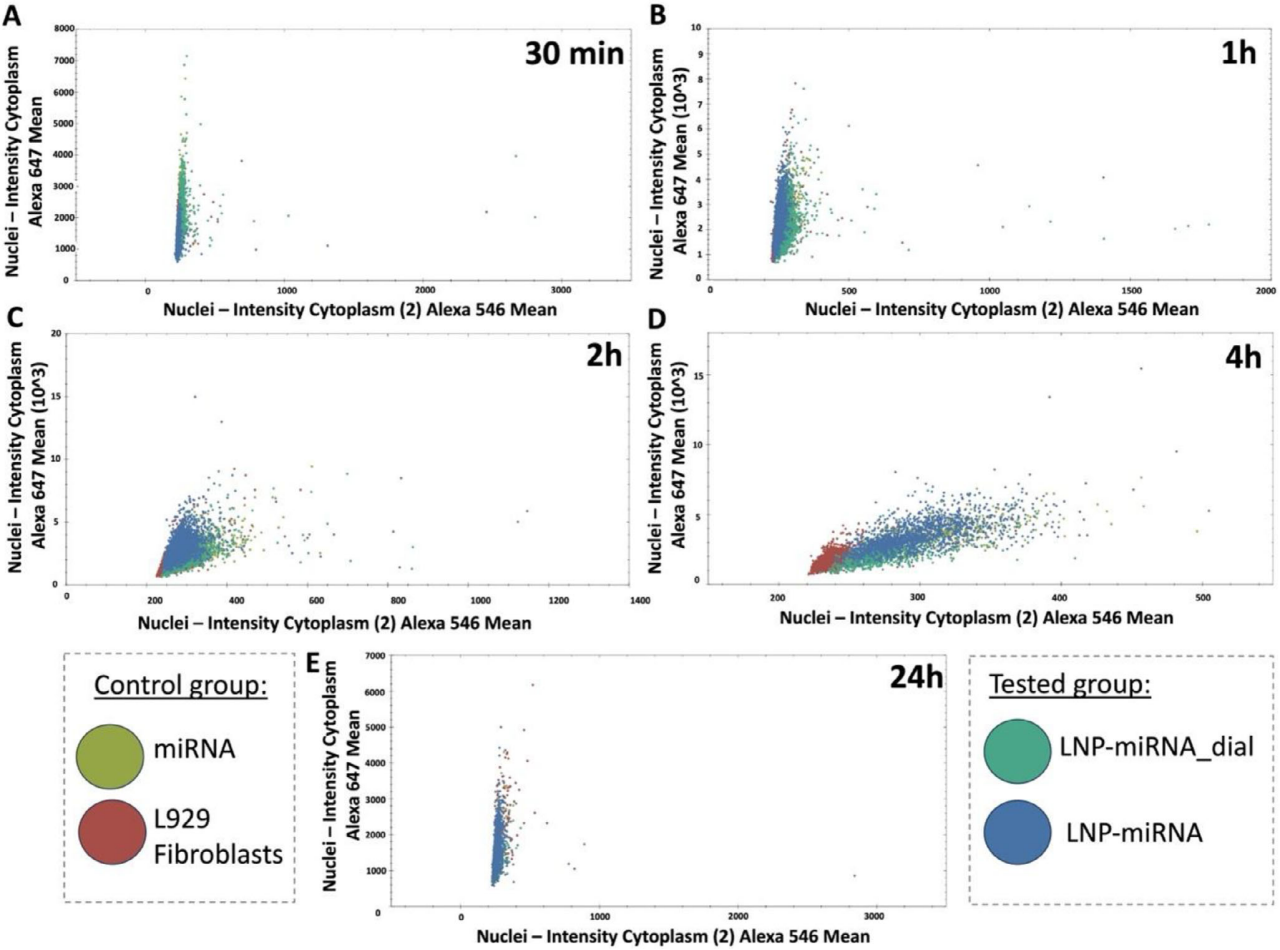
Single‐cell correlation analysis of intracellular miRNA (Cy3, Alexa 546) and LysoTracker (Alexa 647) signal intensity in L929 fibroblasts treated with free miRNA (green), LNP‐miRNA (turquoise), or LNP‐miRNA_dial (blue) at different time points (30 min–24 h). Each dot represents an individual cell. Untreated L929 fibroblasts (red) are included as a control to define baseline LysoTracker signal and background miRNA fluorescence. The data represent measurements acquired from 16 fields of view per condition, collected from three independent samples per group.

Single‐cell correlation analysis was performed to examine the relationship between cytoplasmic Cy3‐miRNA signal (546 nm) and the accumulation of LysoTracker‐positive acidic intracellular compartments (647 nm) following different delivery strategies (Figure 7A–E). Each data point represents an individual cell analyzed at successive time points (30 min, 1 h, 2 h, 4 h, and 24 h) for free miRNA, LNP‐miRNA_dial, LNP‐miRNA, and untreated L929 fibroblasts, formulations. In untreated L929 control cells, which do not contain exogenous miRNA, a low but non‐zero Cy3 (546 nm) signal was consistently detected. This signal reflects cellular autofluorescence and background fluorescence inherent to quantitative imaging and does not indicate the presence of miRNA. Accordingly, the L929 group serves as a baseline reference for distinguishing nonspecific background signal from formulation‐dependent miRNA‐associated fluorescence. In miRNA‐treated groups, a clear shift toward higher Cy3 intensities was observed, indicating intracellular availability of miRNA. Concomitant changes in the relationship between Cy3‐miRNA and LysoTracker signals over time reveal formulation‐dependent differences in intracellular trafficking. In particular, dialyzed LNPs exhibited earlier Cy3‐miRNA availability with a reduced association with LysoTracker‐positive compartments at intermediate time points compared to non‐dialyzed LNPs, consistent with altered endo‐lysosomal processing. In contrast, non‐dialyzed LNP–miRNA showed a stronger and more persistent association with acidic compartments. Overall, this analysis demonstrates that differences in Cy3‐miRNA/LysoTracker correlations arise from formulation‐dependent intracellular trafficking behavior rather than imaging artifacts or differences in cellular uptake, providing quantitative support for the conclusions drawn from confocal imaging.

Our results support a kinetically controlled model of LNP formation, in which particle size, stability, and biological performance depend not only on mixing conditions but also on mechanical perturbation during post‐processing. Kimura et al. demonstrated that rapid vs. gradual ethanol dilution leads to fundamentally different fusion pathways, even when starting from identical microfluidic synthesis conditions. Our observations are consistent with this model and extend it to widely used downstream processing steps that are typically not considered part of LNP formation [20]. Notably, LNPs exhibiting more favorable physicochemical characteristics immediately after synthesis did not result in superior transfection efficiency. This apparent discrepancy can be rationalized by considering that post‐processing‐induced structural rearrangements may alter membrane organization, payload accessibility, or intracellular trafficking without substantially changing ensemble‐averaged size or surface charge.

## 3 Conclusions

This study successfully demonstrates the application of microfluidic technology for the controlled synthesis of lipid nanoparticles LNPs and LNP‐miRNA complexes with desirable physicochemical properties. The microfluidic approach produced spherical, homogeneous nanoparticles with narrow size distributions and good stability, as confirmed by transmission electron microscopy and dynamic light scattering analyses. The observed increase in particle size from 282.6 ± 5.1 to 405.7 ± 4.0 nm upon miRNA encapsulation validated the successful incorporation of the genetic material.

Our findings reveal that post‐synthesis processing steps significantly influence nanoparticle characteristics. Sonication and filtration enhanced particle stability and homogeneity, while dialysis effectively removed unencapsulated components but altered surface properties and increased conductivity. Despite these physicochemical variations, all formulations demonstrated excellent biocompatibility with nearly 100% cell viability and successful miRNA transfection, as evidenced by cytoplasmic and nuclear localization of miRNA in target cells.

Confocal imaging revealed clear, formulation‐dependent differences in the intracellular fate of miRNA delivered either in free form or via lipid nanoparticles. While all delivery strategies enabled cellular internalization of miRNA, their subsequent intracellular trafficking and persistence within acidic endo‐lysosomal compartments differed markedly over time, indicating that post‐entry processing represents a critical determinant of functional delivery. Moreover, the single‐cell correlation analysis provides quantitative evidence that post‐synthesis processing of LNPs modulates the intracellular fate of delivered miRNA at the level of individual cells. Collectively, the results underscore that intracellular fate and endo‐lysosomal processing should be considered alongside conventional physicochemical parameters when evaluating and optimizing lipid nanoparticle‐based miRNA delivery systems.

In summary, this work highlights intracellular trafficking and endosomal escape as key mechanistic bottlenecks in miRNA delivery, underscoring the need to complement physicochemical characterization with intracellular fate analysis. By integrating microfluidic nanoparticle synthesis with advanced imaging and single‐cell approaches, this study provides a robust mechanistic framework for the rational design and optimization of next‐generation LNP‐based miRNA delivery systems.

This work also identifies critical limitations that require further optimization. The encapsulation efficiency of approximately 50% falls substantially below the desired threshold of >80%, likely due to miRNA interactions with microchannel surfaces or suboptimal mixing dynamics. This limitation is particularly significant when working with precious biological materials like miRNA.

In conclusion, while microfluidic synthesis offers superior reproducibility and process control for LNP production, optimization strategies are essential to minimize molecular loss and enhance encapsulation efficiency. Beyond conventional physicochemical optimization, future research should prioritize formulation and post‐processing strategies that promote efficient intracellular trafficking and endosomal escape. Future research should focus on surface modification of microchannels and fine‐tuning of flow parameters to maximize the therapeutic potential of microfluidic‐produced LNP‐miRNA delivery systems. The promising biocompatibility and transfection results provide a strong foundation for advancing this technology toward clinical applications.

## 4 Experimental Section

### 4.1 Materials

The lipid 1,2‐distearoyl‐sn‐glycero‐3‐phosphocholine (DSPC), the lipid 1,2‐distearoyl‐sn‐glycero‐3‐phosphoethanolamine‐N‐[methoxy‐(poly(ethylene glycol))‐2000] (PEG‐DSPE), and the ionizable cationic lipid 1,2‐dioleoyl‐3‐dimethylammonium‐propane (DODAP) were purchased from Avanti Polar Lipids (Alabaster, AL). miRIDIAN microRNA Mimic Red Transfection Control (Cy3‐labeled microRNA, 5 nmol) was purchased from Dharmacon Reagents. Cholesterol (CHOL), sodium citrate, sodium chloride, HEPES, trichloro(1,1,2,2 H‐perfluorooctyl) silane, and citrate buffer were purchased from Sigma–Aldrich (St. Louis, MO, USA). Ethanol and other solvents were obtained from Th.Geyer. Dulbecco's modified Eagle's medium (DMEM), fetal bovine serum (FBS), penicillin‐streptomycin (PS), and EDTA‐trypsin were purchased from Gibco Invitrogen in the USA. The PrestoBlue reagent was acquired from Thermo‐Fisher Scientific, USA. Sylgard 184 was purchased from Dow Corning, USA. L929 murine fibroblast cell line (cat. no. 400260) was obtained from Cytion. The manufacturer guarantees that the cells are free from contamination. Microscopic observation confirmed that no mycoplasma was detected.

### 4.2 Methods

#### 4.2.1 Preparation of PDMS Microfluidic Channels

The microfluidic devices were fabricated using a combination of maskless photolithography (fabrication of the master mold) and soft lithography techniques (fabrication of PDMS replicas). Briefly, the channel designs were created in AutoCAD (Autodesk Inc.), transferred to KLayout (free software by Matthias Köfferlein, https://www.klayout.de), and exported in GDSII format for compatibility with the maskless aligner software. A 3‐inch silicon wafer was coated with the negative photoresist AZ125nXT‐10B (MicroChemicals GmbH, Germany) using a G3P 8 spin coater (Specialty Coating Systems Inc., USA). To obtain a 50 µm‐thick photoresist layer, 3 mL of photoresist was dispensed onto the center of the silicon wafer, followed by spinning at 300 rpm for 5 s with an acceleration of 100 rpm/s (spread step). This was followed by a spin step for thickness definition at 1550 rpm for 12 s with an acceleration of 300 rpm/s. In the final step, the wafer was stopped with a deceleration of 300 rpm/s. The coated wafer was then pre‐heated and soft‐baked on hot plates at 65°C for 3 min and 110°C for 6 min, respectively. These stages ensure homogeneous evaporation of the solvent, and it is ready for exposure. The channel geometry (width = 40 µm, height = 50 µm), was then directly patterned into the photoresist layer using a µMLA maskless aligner with a dose of 4000 mJ/cm^2^ (Heidelberg Instruments, Germany). Following, post‐exposure baking at 120°C for 1 min and development in AZ726 MIF developer (MicroChemicals GmbH, Germany) to obtain the master mold (Figure S1).

To fabricate PDMS microfluidic devices, 30–40 g of polydimethylsiloxane base and curing agent (Sylgard 184) were mixed at a 10:1 (w/w) ratio and degassed in a vacuum chamber. In parallel, the surface of the master mold was modified by keeping under pressure 20 mbar for 10 min in the desiccator with 15 µl trichloro(1,1,2,2 H‐perfluorooctyl) silane (Sigma–Aldrich, USA) to avoid permanent adhesion of PDMS. Then PDMS mixture was poured onto the silanized master mold and cured overnight at 65°C. The wafer was peeled off, then the PDMS chips were cut and punched to create inlet/outlet ports using a biopsy punch (0.75 mm in diameter). The PDMS layer was subsequently activated by low‐pressure oxygen plasma in a Zepto automated plasma cleaner (Diener Electronics GmbH, Germany) with the following conditions: 0.5 W Power, 0.5 sccm Oxygen Flow Rate, 0.2 mbar Pressure. The activated surface was put in contact with the cleaned microscope slide. Flexible microfluidic tubing (PTFE tubing with an internal diameter of 0.80 mm, matching the punched port size and a 20G cut and blunt needles) was inserted into the ports to establish fluidic connections. For a secure and leak‐free fit, the tubing was gently press‐fitted into the punched holes. The external ends of the tubing were connected to syringes. Care was taken to avoid kinks or air bubbles within the tubing, and all connections were checked for leaks before initiating fluid flow. A schematic overview of the microfluidic channel fabrication process, combining maskless photolithography for master mold fabrication and soft lithography for PDMS replica production and device assembly, is presented in Figure S1.

#### 4.2.2 Preparation of Lipid Nanoparticles (LNP) and Lipid Nanoparticles With miRNA‐Cy3 (LNP‐miRNA)

Lipid nanoparticles (LNPs) were prepared using a microfluidic mixing technique. A lipid mixture composed of DODAP, DSPC, cholesterol, and DSPE‐PEG (10:49:40:1 molar ratio) was dissolved in ethanol (organic phase) (10 mg lipids/mL ethanol) and rapidly mixed with an acidic aqueous phase (25 mM sodium acetate, pH 4.0) containing or not microRNAs (LNP‐miRNA or LNP, respectively). The aqueous phase acidity ensured protonation of the ionizable lipid DODAP (pKa ∼6.7), facilitating electrostatic interactions with negatively charged cargo. Mixing was performed in a T‐junction microfluidic channel, with flow rates systematically optimized to 600 µL/min (aqueous) and 200 µL/min (organic) to achieve uniform nanoparticle size and miRNA encapsulation.

#### 4.2.3 LNP Post‐Production Processing Methods

The synthesized lipid nanoparticles (LNPs and LNP‐miRNA) were subjected to sequential post‐production processing steps to refine their physicochemical properties. For the measurements, samples labeled “100%” referred to undiluted LNP/LNP‐miRNA, and “25%” indicated ones diluted with ultrapure water at a 1:3 ratio. Filtration (“F”) was performed using a 0.2 µm sterile SFCA membrane filter (Nalgene, Bionovo) to eliminate microbial contaminants and aggregates exceeding 200 nm, a prerequisite for sterile cell culture applications. Sonication (“S”) followed, utilizing a Cell Ultrasonic Liquid Processor (Sonics & Materials, Inc., USA) with pulsed cycles (2 s ON followed by 2 s OFF) for 2 min on ice to disrupt particle aggregates while mitigating heat‐induced degradation. Post‐sonication, samples were equilibrated to room temperature (22°C–25°C). Dialysis (“dial”) involved a two‐phase protocol: initial purification in 20 mM citrate buffer (pH 4.0, 3.5 kDa MWCO, 1 h) to remove residual ethanol, followed by neutralization in HEPES‐buffered saline (HBS: 20 mM HEPES, 145 mM NaCl, pH 7.4) for 12–18 h [37]. Unencapsulated miRNA was subsequently separated via ultracentrifugation (12 000 rpm, 10 min, 4°C). Finally, thermal incubation (“Heat”) at 36°C for 24 h simulated physiological stability under cell culture conditions. Collectively, these steps (filtration, sonication, dialysis, and thermal treatment) highlight the delicate balance between controlling particle size distribution, ensuring sterility, and maintaining miRNA payload integrity, all of which are essential for optimizing LNPs as effective delivery vehicles in in vitro applications.

#### 4.2.4 Dynamic Light Scattering (DLS)

The particle size distribution and polydispersity index (PDI) were determined using dynamic light scattering (DLS), while the zeta potential, electrophoretic mobility, and conductivity were measured via microelectrophoresis using the Smoluchowski approximation. The analysis was performed using a Zetasizer Nano‐ZS90 (Malvern Instruments, Worcestershire, UK). The colloidal systems were equilibrated for 24 h at 25°C, and all measurements were performed in 3 rounds (10 measurements/round for DLS and PDI, 12 measurements/round for zeta potential).

#### 4.2.5 Scanning Transmission Electron Microscopy (STEM)

Both LNP and LNP‐miRNA were imaged with SEM (Crossbeam 350, Zeiss, Germany) equipped with a STEM detector used in bright field mode (EHT = 20 kV, Working Distance = 2 mm, Aperture Size = 30 µm). Prior to observation, both samples were room temperature dried and gold‐coated on a Cu‐TEM grid to prevent charging, enhance image contrast, and protect the delicate lipid structures during imaging.

#### 4.2.6 Quantification of miRNA Encapsulation Efficiency

miRNA loading within LNPs was quantified via spectrophotometric analysis. Formulation aliquots were dissolved in methanol or ultrapure water, diluted 1:9 (v/v, final volume 0.5 mL), and centrifuged (12 000 rpm, 10 min, 4°C). The supernatant was discarded, and the pellet was resuspended in 0.5 mL of water or methanol. After repeating the centrifugation step, both the final supernatant and resuspended pellet (0.2 mL/well) were analyzed spectrophotometrically. Fluorescence measurements were conducted using a SpectraMax iD3 multiwell plate reader (Molecular Devices, USA). 200 µL of each sample was applied into individual wells of a µ‐Plate 96 Well Square Glass Bottom plate with black walls (Ibidi GmbH, Germany). Excitation was performed at a wavelength of 540 nm, and emitted fluorescence was detected at 580 nm. All measurements were carried out in bottom‐read mode.

(1) $$Bound\;miRNA=\left(LNP-miRNA\;\mathrm{pellet}\right)\;-\left(LNP\;\mathrm{pellet}\right)$$

(2) $$\begin{array}{ccc} Unbound\;miRNA & = & \left(LNP-miRNA\;\mathrm{supernatant}\right)\;\\ & & -\left(LNP\;\mathrm{supernatant}\right)\; \end{array}$$

Encapsulation efficiency (%) was derived using:

(3) $$\begin{array}{ccc} Binding\;Efficiency\;\left(\%\right) & = & \frac{\mathrm{Bound}\;\mathrm{miRNA}}{\mathrm{Bound}\;\mathrm{miRNA}+\;\mathrm{Unbound}\;\mathrm{miRNA}}\\ & & \;*100\%\; \end{array}$$

Sample absorbance values were normalized by applying a 10X dilution factor correction. Results represent the mean of triplicate measurements across three independent batches (n = 3).

#### 4.2.7 Preparation of Glass Microfluidic Channels

Microfluidic channels were fabricated directly in soda‐lime glass substrates using ultrafast laser micromachining. Channel geometries were designed in AutoCAD and exported in a vector format compatible with the laser control software. Prior to laser processing, glass substrates were cleaned by sequential rinsing in acetone, isopropanol, and deionized water, followed by drying under a nitrogen stream. Laser structuring was carried out using a 20 W Jasper X0 laser system (Fluence Ltd.) operating at a central wavelength of 1030 nm. The laser delivered ultrashort pulses and was integrated with a galvanometric scanning system (IntelliScan14, SCANLAB LTD) for precise beam positioning. The laser beam was focused onto the glass surface using an F‐theta scanning lens mounted on the galvanometric head. Processing parameters, including pulse energy (25%), repetition rate, scanning speed, and hatch spacing (5 µm), were optimized to obtain microchannels with depths of approximately 50 µm while maintaining smooth channel walls and minimizing cracking or thermal damage. Material removal was achieved through nonlinear absorption in the soda‐lime glass, enabling localized ablation despite the material's transparency at 1030 nm. Microchannels were formed by multiple raster scans along the predefined paths to ensure uniform channel depth and width across the entire structure. After laser ablation, the structured glass substrates were cleaned in an ultrasonic bath filled with deionized water to remove debris and redeposited material. The samples were subsequently rinsed with isopropanol and dried. Prior to bonding, the glass was cleaned with Alconox 1%. Enclosed microfluidic devices were formed by bonding the structured glass substrate to an unstructured soda‐lime glass. The experimental procedure followed the protocol developed by Allen and Chiu [38]. After cleaning, both glass substrates were rinsed with a 0.5% Alconox and 0.5% calcium acetate aqueous slurry, which was briefly trapped between the surfaces to ensure full contact. The substrates were rinsed in flowing deionized water, separated, and reassembled under the water stream in a V‐shaped configuration, taking care to avoid air bubble entrapment. Excess water was gently blotted with lint‐free wipes, and the glass assembly was clipped between microscope slides and dried at 60°C for 1–2 h. Final thermal bonding was performed at 115°C for 2 h after inspection confirmed the absence of defects. Inlet and outlet ports were fabricated by laser drilling at positions corresponding to the ends of the microchannels. PTFE tubing with an internal diameter matched to the port size was inserted into the openings by press‐fitting and gluing, and then connected to syringe‐based flow control. All fluidic connections were inspected to ensure leak‐free operation prior to experiments.

#### 4.2.8 LNP‐miRNA Integrity After Post‐Processing

To evaluate the impact of post‐processing methods on the integrity of miRNA within LNPs, fluorescence signals of LNP–miRNA formulations were compared before and after different treatments. Untreated samples were analyzed immediately after preparation (non‐treated/0 h). Sonication was performed using a Cell Ultrasonic Liquid Processor (Sonics & Materials, Inc., USA) with pulsed cycles (2 s ON followed by 2 s OFF) for 2 min on ice to disrupt particle aggregates while minimizing heat‐induced degradation (sonicated/0 h). Thermal treatment was carried out by incubating samples at 36°C for 24 h to simulate physiological stability under cell culture conditions (heated/24 h). In addition, combined sonication followed by heating was applied (sonicated/heated/24 h). For fluorescence measurements, a SpectraMax iD3 multiwell plate reader (Molecular Devices, USA) was used, and 200 µL of each sample was dispensed into individual wells of a µ‐Plate 96 Well Square Glass Bottom plate with black walls (Ibidi GmbH, Germany). Excitation was performed at 540 nm, and fluorescence emission was detected at 580 nm. All measurements were carried out in bottom‐read mode.

### 4.3 In Vitro Study

#### 4.3.1 Cell Proliferation

L929 murine fibroblast purchased from Sigma–Aldrich was cultured in DMEM (high glucose medium) supplemented with 10% FBS and 1% P/S at 37°C in 5% CO_2_ atmosphere. When the confluence of cells reached approximately ∼80%, the cells were collected using 0.05% EDTA‐trypsin and centrifuged at 1200 rpm for 5 min. The cell suspension was diluted in the culture media to attain a seeding density of 15 000 cells per 1 well (96‐well plate) for the proliferation test. The cells were treated with sterile solutions (through a 0.2 µm filter) of LNP (10, 20, 30, 40 µL), LNP‐miRNA (10, 20, 30, 40 µL), miRNA (10, 20, 30, 40 µL), and CTRL, which do not include additives to the medium. Each group had 5 replicates. The proliferation was measured with the PrestoBlue assay on days 1 and 3 to obtain a quantitative assessment of the samples' biocompatibility. The cells treated with LNP, LNP‐miRNA, miRNA, and CTRL were treated with a solution of 10% (v/v) PrestoBlue reagent in culture medium and incubated for 2 h at 37°C and 5% CO_2_. After that, triplicates of each 100 µL aliquot of the PrestoBlue solution were transferred to a 96‐well plate and analyzed at excitation 530 nm and emission at 620 nm by using a fluorometer plate reader (Fluoroskan Ascent TM Microplate Fluorometer, Thermo Scientific, USA).

#### 4.3.2 Calcein and Hoechst Staining

To enable accurate enumeration of individual cells, cells were seeded at a low density of 500 cells per well in 96‐well plates (n = 5 replicates per group) and allowed to adhere for 24 h. Afterward, 10 µL of sterile test formulations were added: free miRNA (miRNA), blank LNPs (LNP), dialyzed blank LNPs (LNP_dial), miRNA‐loaded LNPs (LNP‐miRNA), dialyzed miRNA‐loaded LNPs (LNP‐miRNA_dial), or untreated controls (CTRL). After 24 h of treatment, cells were stained with Calcein‐AM and Hoechst to quantify live cells and nuclei, respectively. Calcein‐AM (1 mg/mL stock) was diluted 1:1000 (1 µL/well final concentration) in complete medium and incubated for 15 min at 36°C. Hoechst was added at a 1:5000 dilution and incubated for 10 min at 36°C, followed by gentle rinsing with PBS. Live‐cell fluorescence was acquired using the Operetta CLS high‐content imaging system (PerkinElmer), with filter settings for Calcein (Ex/Em: 488/520 nm) and Hoechst (Ex/Em: 350/461 nm). Fluorescence quantification was performed using integrated image analysis software to evaluate cell viability and total cell number. Importantly, the excitation/emission spectra of Calcein and Hoechst did not overlap with the Cy3 channel (Ex/Em: 550/570 nm), allowing for potential parallel use in experiments involving Cy3‐labeled miRNA without spectral interference.

#### 4.3.3 L929 Cell Internalization

The spatial relationship between intracellular miRNA and the nuclei of L929 cells was analyzed using live‐cell imaging. Cells were plated in 96‐well plates at 10 000 cells per well and allowed to adhere overnight. Treatments were performed the following day using free Cy3‐tagged miRNA, Cy3‐labeled miRNA encapsulated in lipid nanoparticles, or dialyzed miRNA‐loaded LNPs, applied in a total volume of 10 µL per well. Nuclear staining was achieved by co‐incubation with Hoechst dye (5 µM). After a short incubation period of 15 min, fluorescence imaging was carried out on an Operetta CLS high‐content imaging platform (PerkinElmer). miRNA and nuclear signals were detected using the Cy3 (Ex/Em ≈ 550/570 nm) and Hoechst (Ex/Em ≈ 350/461 nm) channels, respectively. Images were acquired at 30 min, 1 h, 2 h, 4 h, and 24 h following treatment, enabling temporal evaluation of intracellular miRNA distribution relative to the nucleus.

#### 4.3.4 Endo‐Lysosomal Trafficking and Endosomal Escape of miRNA

To investigate the endosomal escape of lipid nanoparticles, L929 cells were seeded at a density of 10 000 cells per well in 96‐well plates and incubated overnight. The following day, cells were transfected with Cy3‐labeled miRNA (miRNA), Cy3‐labeled miRNA‐loaded lipid nanoparticles (miRNA‐LNP), and dialyzed miRNA‐LNP (miRNA‐LNP_dial) at a final volume of 10 µL per well. Untreated L929 cells, without any treatment, served as the control group. Simultaneously, LysoTracker Deep Red (50 nM) and Hoechst (5 uM) were added to the cells. After 15 min of incubation, L929 cells were imaged using the Operetta CLS high‐content imaging system (PerkinElmer). Three detection channels were used: Cy3 (Ex/Em ∼550/570 nm) to visualize labeled miRNA, Alexa Fluor 647 (Ex/Em ∼ 647/668 nm) to label acidic organelles, like lysosomes, and Hoechst (Ex/Em ∼ 350/461 nm) to label nuclei of the cells. Images were taken at 5 time points: 30 min, 1 h, 2 h, 4 h, and 24 h. Overlaying Cy3 and Alexa Fluor 647 images enabled the assessment of miRNA distribution within cells and the evaluation of nanoparticle‐mediated delivery dynamics.

### 4.4 Statistical Analysis

The data underwent statistical analysis using a one‐way ANOVA followed by Tukey's multiple pairwise comparisons test, calculated by OriginPro software. Significance was set at the 0.05 level. Symbols indicate significance (^****^ *p* <0.0001, ^***^ *p* <0.001, ^**^ *p* <0.01, ^*^ *p* <0.05). For DLS measurements, reported values represent the mean ± standard deviation of three repeated measurements (n = 3) performed for each condition on the same nanoparticle formulation.

## Funding

SONATA BIS Project No. 2020/38/E/ST5/00456 supported by the National Science Centre (NCN). A.K.K acknowledges the support of NCN under SONATA Project No. 2024/55/D/ST5/01357.

## Conflicts of Interest

The authors declare no conflicts of interest.

## Supporting information




**Supporting File**: advs74276‐sup‐0001‐SuppMat.docx.

## Data Availability

The data that support the findings of this study are available from the corresponding author upon reasonable request.
