# Supplementary material for: Microfluidic‐Driven Lipid Nanoparticles for Improved miRNA Delivery via Endo‐Lysosomal Trafficking Optimization
Source: Adv Sci (Weinh). 2026 Feb 6;13(24):e19225. doi: 10.1002/advs.202519225 (PMC13115942; doi:10.1002/advs.202519225)
Supplement: Supplementary file 1 — Supporting File: advs74276‐sup‐0001‐SuppMat.docx. [file ADVS-13-e19225-s001.docx]

Supporting Information

****Microfluidic-Driven Lipid Nanoparticles for Improved miRNA Delivery via Endo-Lysosomal Trafficking Optimization****

Alicja Kosik-Kozioł, Michał Pruchniewski, Daniel Rybak, Piotr Jenczyk, Karolina Zakrzewska, Magdalena Bartolewska, Sławomir Błoński, Paweł Nakielski, Filippo Pierini^*^

Alicja Kosik-Kozioł, Daniel Rybak, Karolina Zakrzewska, Magdalena Bartolewska, Sławomir Błoński, Paweł Nakielski, Filippo Pierini^*^

Department of Biosystems and Soft Matter, Institute of Fundamental Technological Research, Polish Academy of Sciences, Warsaw 02-106, Poland

E-mail: [fpierini@ippt.pan.pl](mailto:fpierini@ippt.pan.pl)

Michał Pruchniewski

Department of Nanobiotechnology, Institute of Biology, Warsaw University of Life Sciences, Warsaw 02-786, Poland.

Piotr Jenczyk

Department of Mechanics of Materials, Institute of Fundamental Technological Research, Polish Academy of Sciences, Warsaw 02-106, Poland

Keywords: miRNAs-Cy3, lipid nanoparticles, microfluidics, endo-lysosomal trafficking, LNP post-processing

***
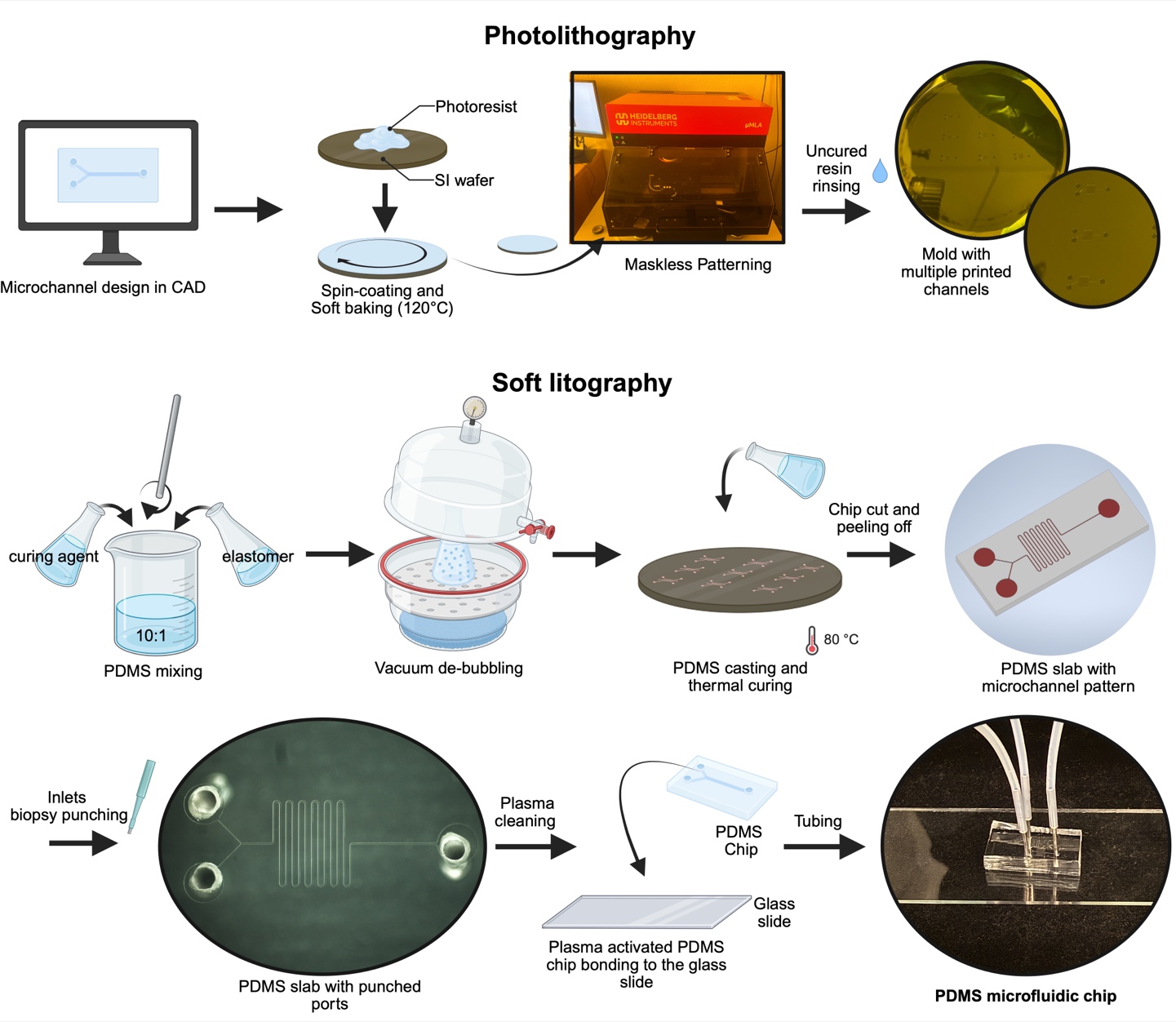
***

**Figure S1.** Schematic overview of microfluidic channel fabrication combining maskless photolithography for master mold fabrication and soft lithography for PDMS replica production and device assembly.

**Figure S2.** Comparison of LNP size distributions as a function of AQUA:OIL flow rate optimization in two different microfluidic channels. Panels **A-H** show size distributions obtained using the PDMS microfluidic channel at various AQUA:OIL flow rate ratios. Panel **I** presents the size distribution obtained using the glass microfluidic channel at an AQUA:OIL flow rate of **600:200 µL/min**, which was identified as the most optimal condition and subsequently applied in cellular experiments and binding efficiency studies.

**Table S1.** Size distribution of LNPs before dialysis.

|  | **-/-** | **S/-** | **S/F** |
| --- | --- | --- | --- |
| **100%** | 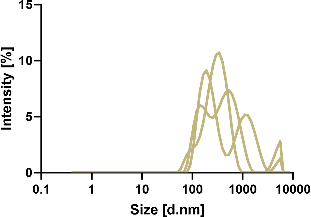 | 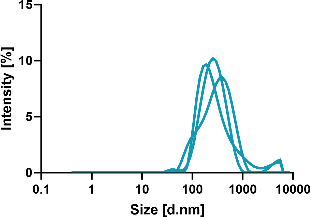 | 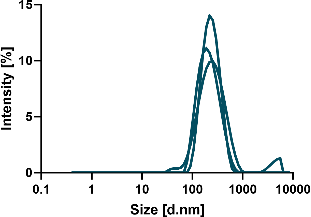 |
| **100% miRNA** | 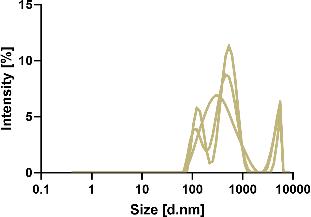 | 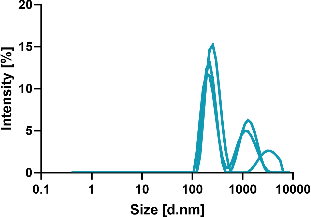 | 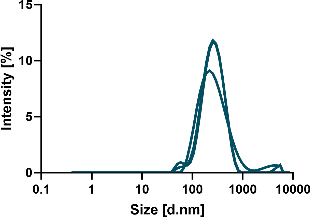 |
| **25%** | 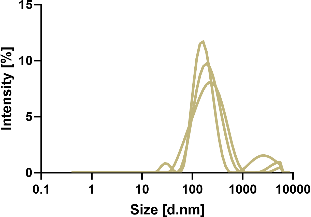 | 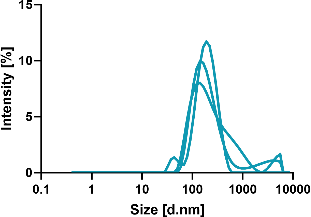 | 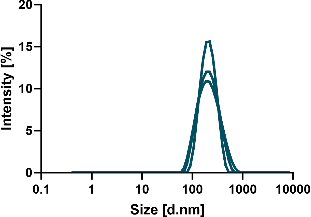 |
| **25% miRNA** | 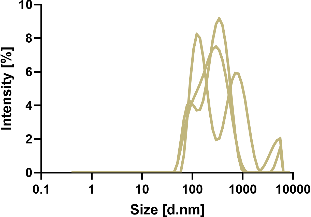 | 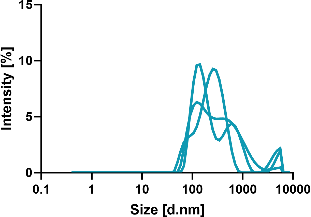 | 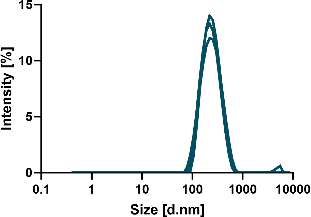 |

**Table S2.** Size distribution of LNPs after dialysis.

|  | **-/-** | **S/-** | **S/F** |
| --- | --- | --- | --- |
| **100%** | 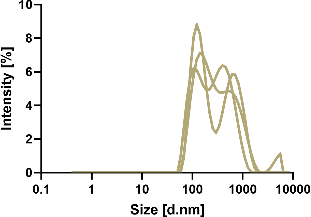 | 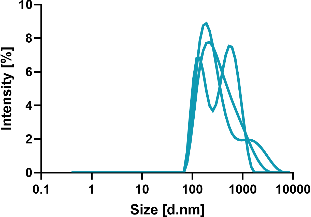 | 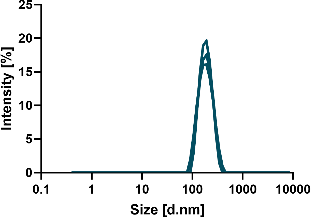 |
| **100% miRNA** | 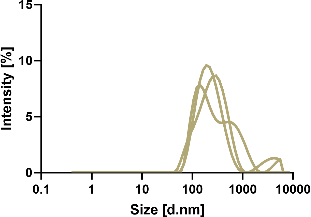 | 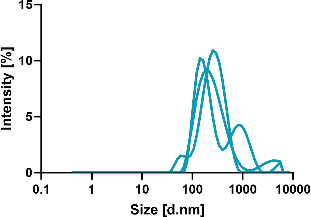 | 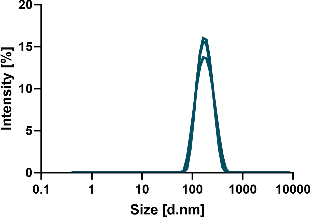 |
| **25%** | 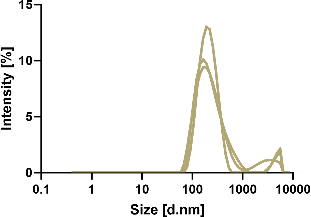 | 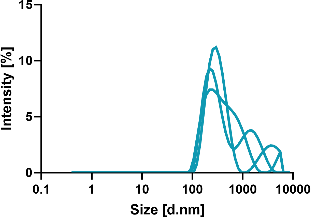 | 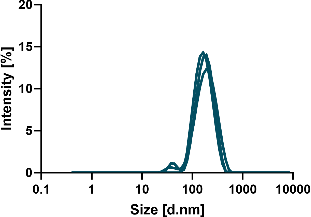 |
| **25% miRNA** | 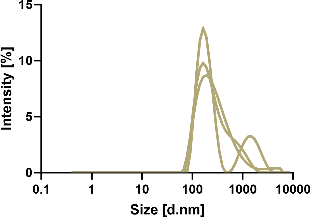 | 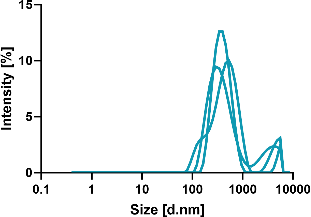 | 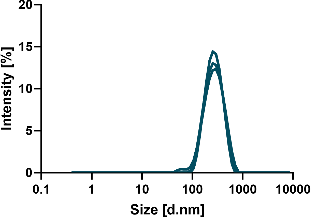 |

**Table S3.** Size distribution of LNPs after heating at 37°C.

|  | **-/Heat** | **Dialysis/Heat** |
| --- | --- | --- |
| **100%** | 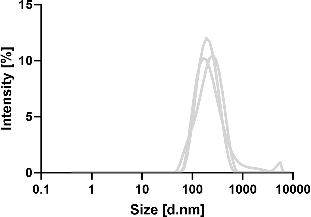 | 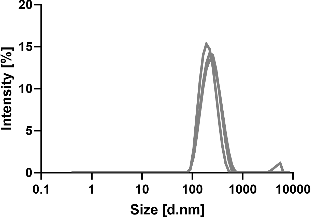 |
| **100% miRNA** | 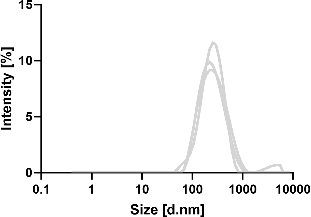 | 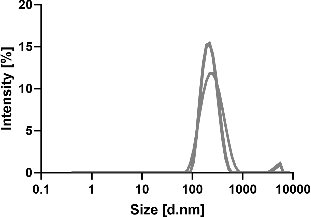 |
| **25%** | 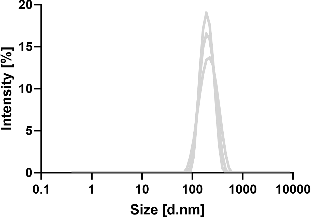 | 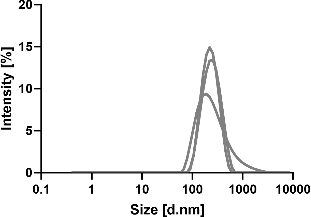 |
| **25% miRNA** | 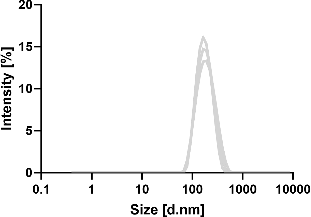 | 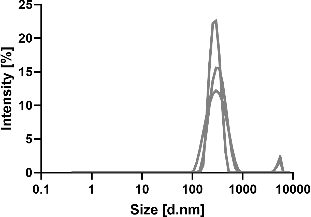 |


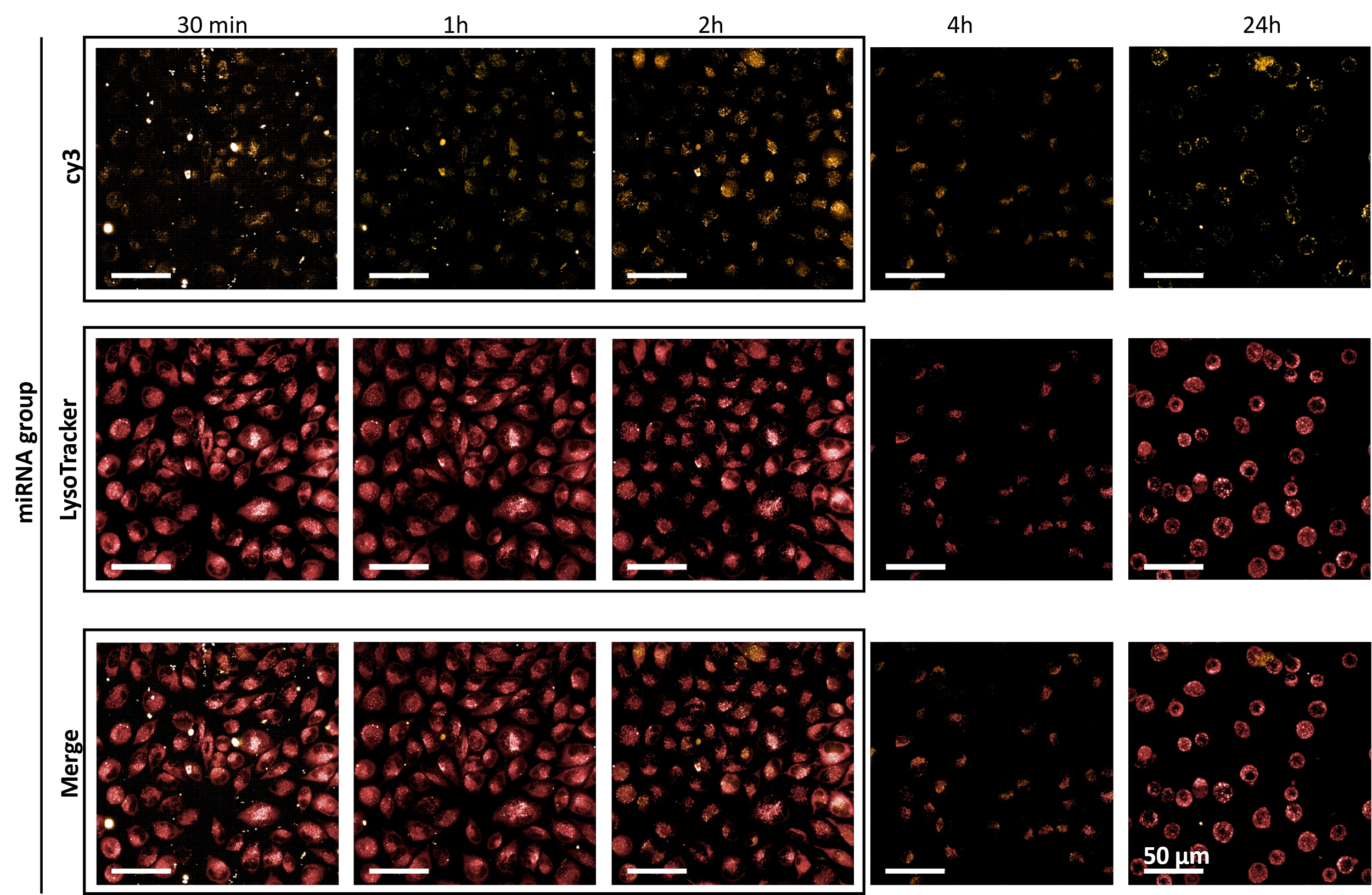


**Figure S3.** Time-dependent intracellular localization of Cy3-labeled miRNA and LysoTracker signal in the miRNA group. Representative fluorescence images were acquired at 30 min, 1 h, 2 h, 4 h, and 24 h post-treatment. Images at 30 min, 1 h, and 2 h represent time-lapse imaging of the same field of cells, as live-cell imaging with LysoTracker is recommended by the manufacturer for up to 2 h. The Cy3 channel shows the miRNA signal, LysoTracker marks acidic compartments, and merged images illustrate their spatial relationship over time. Scale bar: 50 µm.


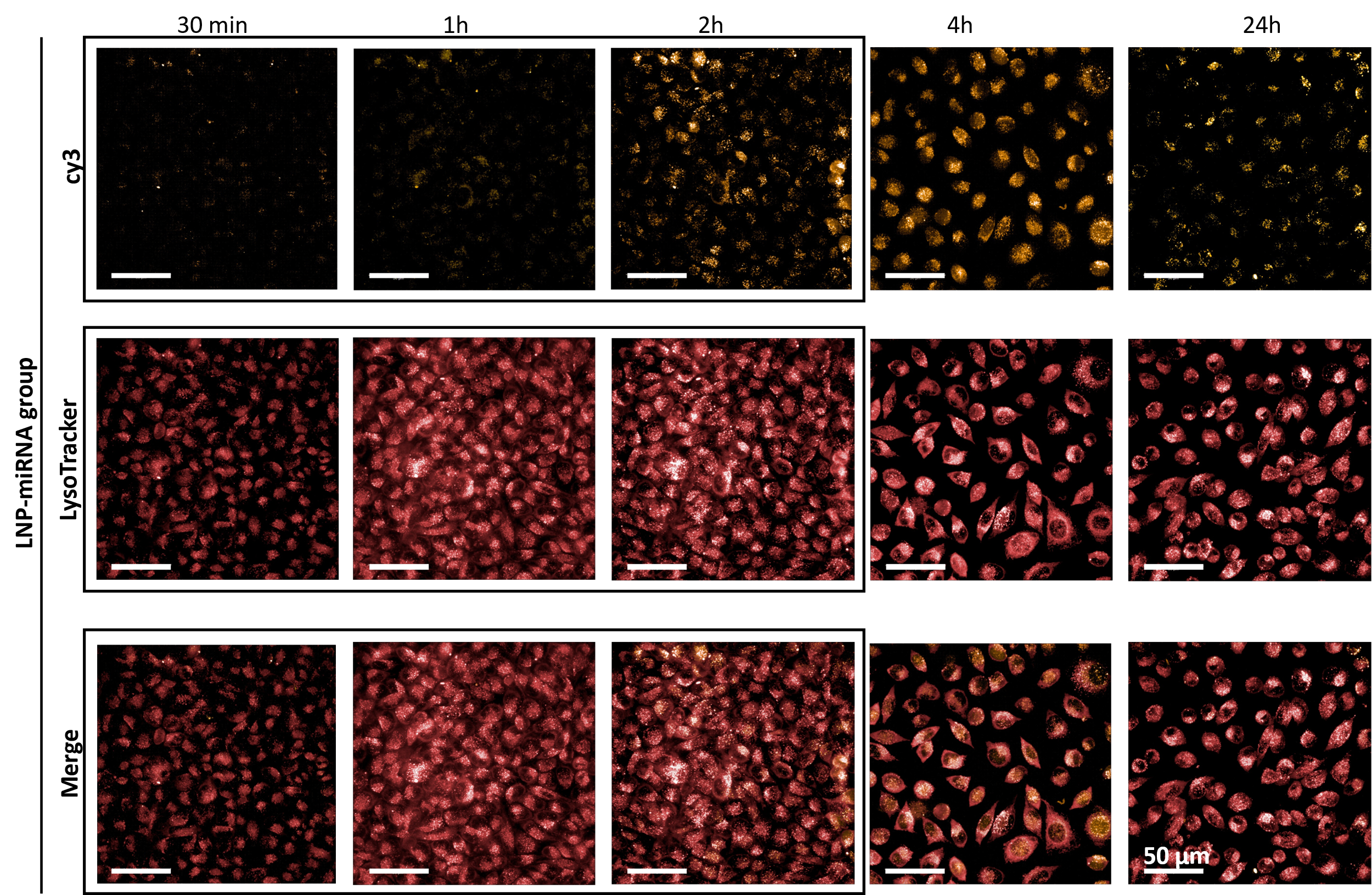


Figure S4. Time-dependent intracellular localization of Cy3-labeled miRNA and LysoTracker signal in the LNP-miRNA group. Representative fluorescence images were acquired from 30 min to 24 h post-treatment. Images from 30 min to 2 h represent time-lapse imaging of the same field of cells. The Cy3 channel shows the miRNA signal, LysoTracker marks acidic compartments, and merged images illustrate their spatial relationship over time. Scale bar: 50 µm.


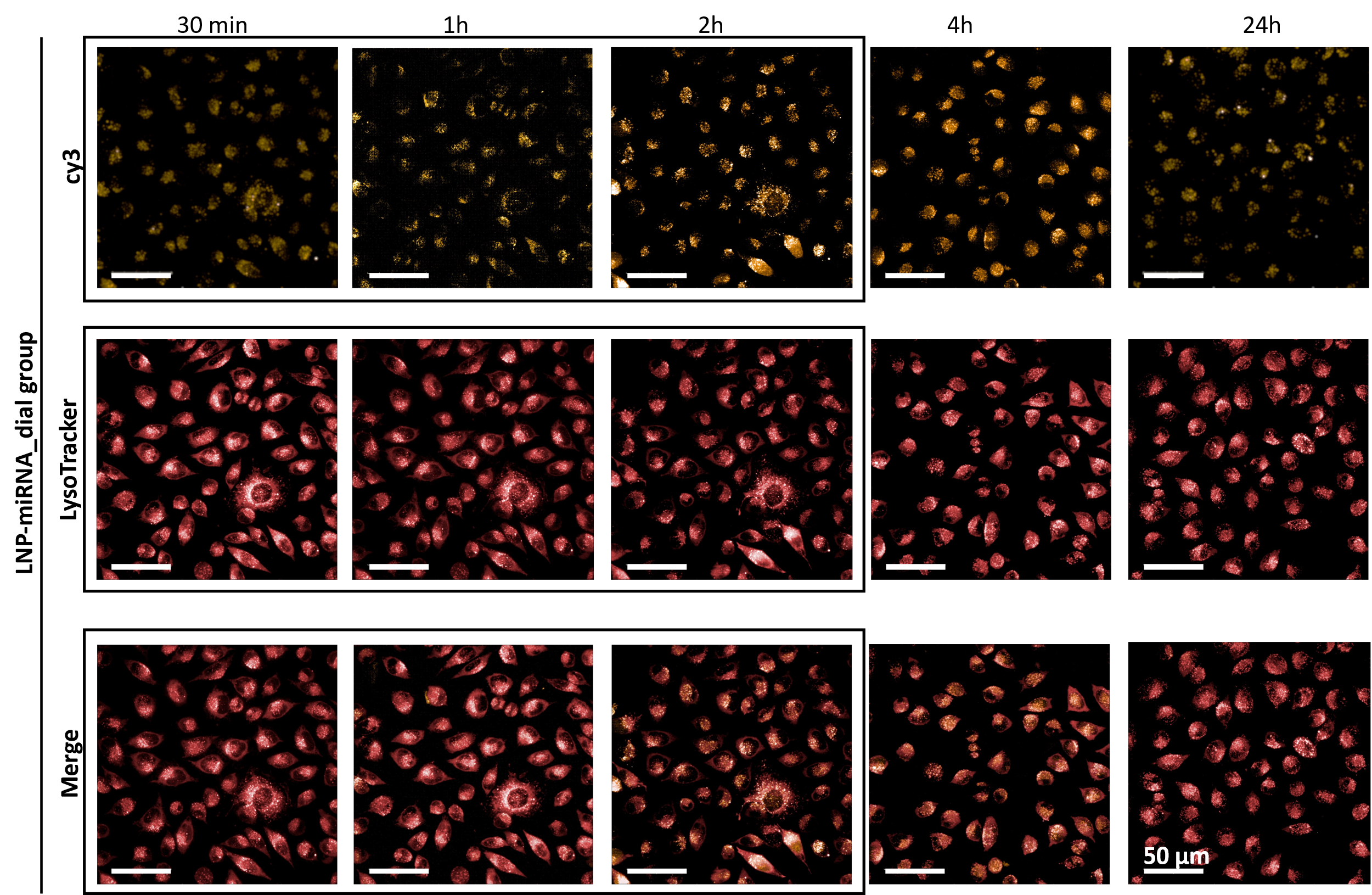


Figure S5. Time-dependent intracellular localization of Cy3-labeled miRNA and LysoTracker signal in the LNP-miRNA_dial group. Representative fluorescence images were acquired from 30 min to 24 h post-treatment. Images from 30 min to 2 h represent time-lapse imaging of the same field of cells. The Cy3 channel shows the miRNA signal, LysoTracker marks acidic compartments, and merged images illustrate their spatial relationship over time. Scale bar: 50 µm.


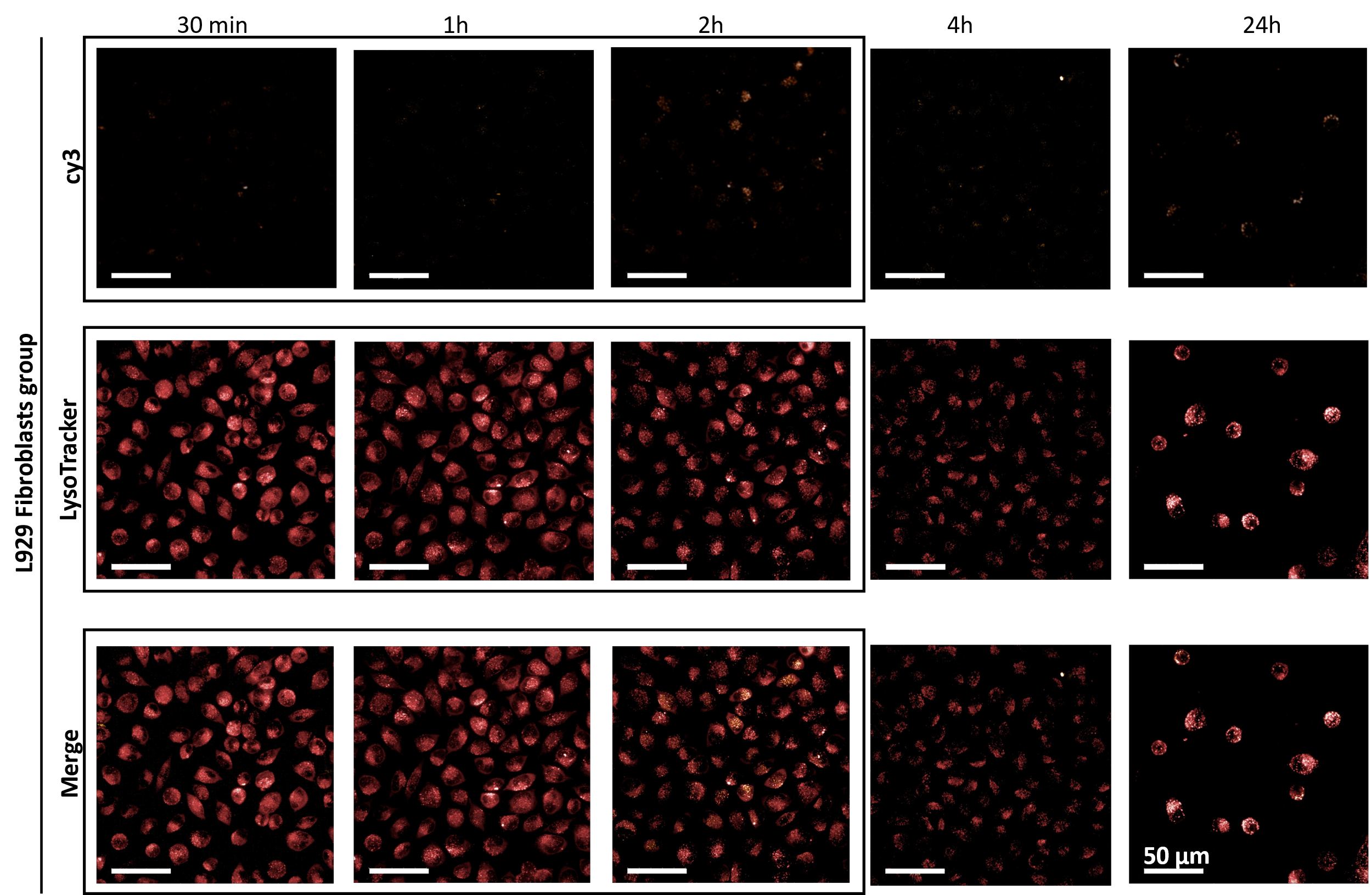


Figure S6. Time-dependent intracellular localization of Cy3-labeled miRNA and LysoTracker signal in the control L929 Fibroblasts group. Representative fluorescence images were acquired from 30 min to 24 h post-treatment. Images from 30 min to 2 h represent time-lapse imaging of the same field of cells. The Cy3 channel shows the miRNA signal, LysoTracker marks acidic compartments, and merged images illustrate their spatial relationship over time. Scale bar: 50 µm.
